# Inference of Cell Mechanics in Heterogeneous Epithelial Tissue Based on Multivariate Clone Shape Quantification

**DOI:** 10.3389/fcell.2017.00068

**Published:** 2017-08-03

**Authors:** Alice Tsuboi, Daiki Umetsu, Erina Kuranaga, Koichi Fujimoto

**Affiliations:** ^1^Laboratory of Theoretical Biology, Department of Biological Sciences, Osaka University Toyonaka, Japan; ^2^Laboratory of Histogenetic Dynamics, Graduate School of Life Sciences, Tohoku University Sendai, Japan

**Keywords:** cell mechanics, PCA, heterogeneity, tumor, cell sorting, cell mixing, *Drosophila*, vertex model

## Abstract

Cell populations in multicellular organisms show genetic and non-genetic heterogeneity, even in undifferentiated tissues of multipotent cells during development and tumorigenesis. The heterogeneity causes difference of mechanical properties, such as, cell bond tension or adhesion, at the cell–cell interface, which determine the shape of clonal population boundaries via cell sorting or mixing. The boundary shape could alter the degree of cell–cell contacts and thus influence the physiological consequences of sorting or mixing at the boundary (e.g., tumor suppression or progression), suggesting that the cell mechanics could help clarify the physiology of heterogeneous tissues. While precise inference of mechanical tension loaded at each cell–cell contacts has been extensively developed, there has been little progress on how to distinguish the population-boundary geometry and identify the cause of geometry in heterogeneous tissues. We developed a pipeline by combining multivariate analysis of clone shape with tissue mechanical simulations. We examined clones with four different genotypes within *Drosophila* wing imaginal discs: wild-type, *tartan* (*trn*) overexpression, *hibris* (*hbs*) overexpression, and *Eph* RNAi. Although the clones were previously known to exhibit smoothed or convoluted morphologies, their mechanical properties were unknown. By applying a multivariate analysis to multiple criteria used to quantify the clone shapes based on individual cell shapes, we found the optimal criteria to distinguish not only among the four genotypes, but also non-genetic heterogeneity from genetic one. The efficient segregation of clone shape enabled us to quantitatively compare experimental data with tissue mechanical simulations. As a result, we identified the mechanical basis contributed to clone shape of distinct genotypes. The present pipeline will promote the understanding of the functions of mechanical interactions in heterogeneous tissue in a non-invasive manner.

## Introduction

There are intrinsic differences among cells within any population, even in genetically uniform populations such as, clonal populations of bacteria, yeasts, and undifferentiated plant and animal cells (Elowitz, 2002; Raser, 2004; Raj and van Oudenaarden, 2008; Eldar and Elowitz, 2010; Itzkovitz et al., 2011; Meyer and Roeder, 2014). The non-genetic (isogenic) heterogeneity stems from intrinsic noise due to stochastic fluctuations in gene expression and extrinsic noise due to stochastic changes in upstream signal transduction (Paulsson, 2004; Shibata and Fujimoto, 2005). Theoretical and experimental discrimination between intrinsic and extrinsic noise (Elowitz, 2002; Swain et al., 2002) promoted an understanding of not only the molecular mechanisms but also the functional significance of the non-genetic heterogeneity (Raj and van Oudenaarden, 2008; Eldar and Elowitz, 2010). Genetic heterogeneity in tissues arises from spontaneous mutations in cell lineages. The emergence of cellular heterogeneity in epithelial tissues alters the morphology of the boundaries between neighboring populations and thereby affects the cellular geometry in the tissue. Alterations of the geometrical cellular configuration between clonal populations (clones) often have physiological consequences. Clonal segregation caused by Eph receptors has been shown to play a tumor-suppressive role in colorectal cancer by compartmentalizing the cancer cells and thereby limiting their invasion into normal tissues (Cortina et al., 2007; Porazinski et al., 2016). In the context of the cell competition, known as the tissue homeostatic system, to eliminate unfit cells from heterogeneous populations (Vincent et al., 2013; Amoyel and Bach, 2014; Morata and Ballesteros-Arias, 2015), the intermingling of cells at clonal boundaries facilitates the competition by increasing the contact length between competing genotypes, so-called winner and loser cells (Levayer et al., 2015; Levayer and Moreno, 2016). Those insights suggest the potential to predict physiological consequences (e.g., tumor malignancy) based on the quantification of clone shapes. Therefore, the establishment of a pipeline that combines the quantification of clone shapes with an analysis of the physical mechanisms underlying the clone shapes would be beneficial.

A major contributing factor for clone shape is mechanical interactions at the cell–cell interface. Cell–Cell adhesion mediated by adhesion molecules and contractility exerted by the actomyosin network contribute to the tension on the cell–cell interface (Lecuit and Lenne, 2007). The differential adhesion and contractility among genetically heterogeneous cells have been experimentally shown to play a major role in cell segregation by driving or guiding cell rearrangements (Nose et al., 1988; Krieg et al., 2008; Maitre et al., 2012; Maître et al., 2016). The role of such mechanical cell–cell interactions on cell sorting has been also theoretically studied using computer simulations of tissue mechanics (Graner and Glazier, 1992; Brodland, 2002). More recently, experimental evidence in combination with models has shown that the alteration of boundary morphology can be explained by the tissue anisotropy of mechanical tension at the cell–cell interface (Landsberg et al., 2009; Monier et al., 2010; Aliee et al., 2012; Rudolf et al., 2015). The relative strength of such mechanical tension (or stress) has been estimated non-invasively from time lapse of cell shape dynamics (Brodland et al., 2010; Chiou et al., 2012; Ishihara and Sugimura, 2012; Nier et al., 2016) or images of fixed tissue (Brodland et al., 2014), and invasively using physical perturbation (e.g., laser ablation of cell junctions) (Sugimura et al., 2016, and reference therein). Moreover, a recent study showed that the “clone tension,” which is defined by the average strength of the junctional tension inside and on the border of the clone relative to that on the outside, uniquely distinguishes smoothed and convoluted clone shapes (Bosveld et al., 2016a). Hence, reliable quantification of clone shape should make it possible to identify the averaged strength of tensions in heterogeneous tissue.

Although several quantification methods for clone shape, such as, circularity (Milán et al., 2002; Chang et al., 2011) and cell mixing index (Umetsu et al., 2014a; Levayer et al., 2015), have been established, each has been applied only independently. It is not known whether those methods can be applied to any clone shape or are suited for some particular clone shape. Moreover, it has been unknown whether such methods are sufficient to distinguish clone shapes or the other methods (e.g., cell area) are required. The combinatorial use of multiple quantitative methods would more reliably evaluate the clone shapes of various genotypes.

**Figure 1:**
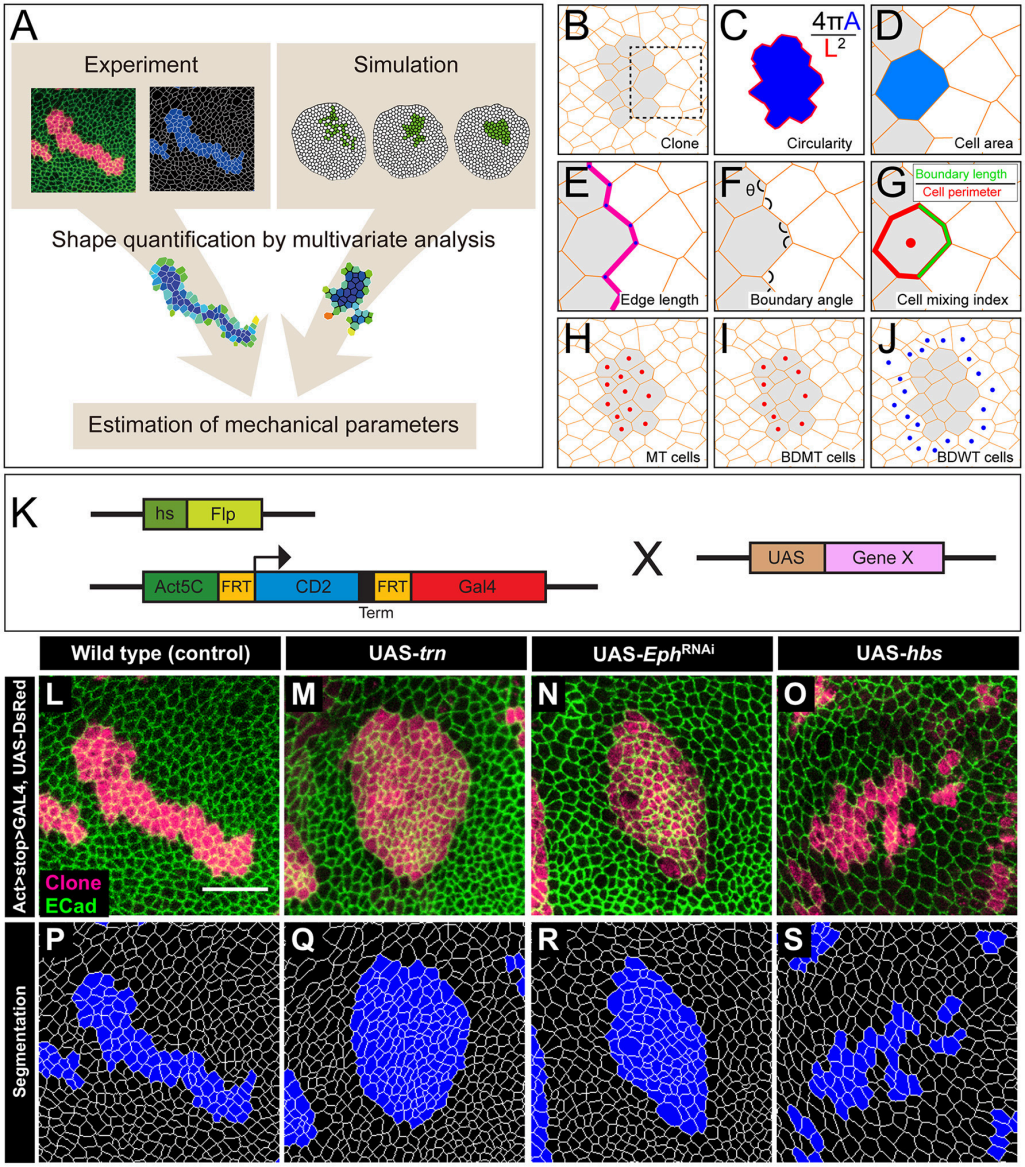
The multivariate estimation pipeline using quantitative evaluation of clone shape. **(A)** Schematic summary of the inference procedure. **(B–J)** Criteria used for the evaluation of clone shape. Circularity **(C)** was measured only for closed clones, while the other six cell-based criteria **(D–J)** were measured for both closed clones and open clones. The cell-based criteria **(D–J)** were calculated for each edge, tri-cellular junction, or cell, and the values were averaged over each clone. **(B)** Scheme for a clone. Orange lines represent adherens junctions, and cells within a clone are shaded. **(C)** Circularity calculated as 4π A/(L^2^), where “A” represents the area of the clone, and “L” is the perimeter length of the clonal interface. The circularity of a perfect circle should be 1. **(D–G)** Magnified images of the box in **(B)**. **(D)** Area of a cell within a clone normalized by that of all wild-type cells surrounding the clone in a disc. **(E)** Length of edges (cell junctions between neighboring vertices) at the clone boundary normalized by that of edges between wild-type cells. **(F)** Boundary angle, which is the smaller angle (<180°) between neighboring junctions along the clone boundary at every tri-cellular junction (three-way vertices). **(G)** Cell mixing index, which is the fraction of the perimeter of a cell that is shared with cells from the other side of the clone boundary. **(H–J)** The cells used to calculate the average cell mixing index for MT, BDMT, and BDWT are marked by red dots in (**H**, all cells within the clone) **(I**, clonal cells beside the clone boundary) and blue dots in **(J**, wild-type cells beside the clone boundary), respectively. **(K)** Scheme for genetic manipulation used to generate the clones. GAL4 is expressed only when the CD2 cassette, which includes a transcription termination sequence and is flanked by FRT sites, is excised upon Flippase (Flp) induction by heat shock. Once expressed, GAL4 binds to the UAS sequence and drives expression of the downstream gene. **(L–O)** Clones expressing a marker for wild-type **(L)**, *trn* **(M)**, double-strand RNA against *Eph* **(N)**, and *hbs* **(O)**. **(P–S)** Segmentation of the cell junctions in **(L–O)**.

In this study, we provide a pipeline to quantify the clone shape difference and identify the cause of the difference by combining a multivariate quantitative analysis of clone shape with computer simulations of tissue mechanics (Figure 1A). Using fixed *Drosophila* wing imaginal discs, we examined four genotypes [wild-type control, *tartan* (*trn*) overexpression, and *Eph* RNAi, *hibris* (*hbs*) overexpression], which exhibit smoothed or convoluted clone morphologies yet have unknown cell mechanical properties. Clones that overexpress *Drosophila trn*, which encodes leucine rich repeat-containing transmembrane proteins (Milán et al., 2001, 2002; Sakurai et al., 2007), adopt a smooth shape. The knockdown of *Eph*, which encodes tyrosine kinases of the Eph receptor protein family, also generates round clones (Umetsu et al., 2014b). In contrast, the overexpression of *hbs*, an immunoglobulin superfamily member, leads to the separation of the *hbs*-overexpressing cells and their partial mixing into surrounding cells (Bao et al., 2010), resulting in a convoluted clone morphology. We used multiple cell-based criteria to quantify the clone morphology. While no single criterion alone was able to distinguish all four genotypes, by combinatorial use of multiple criteria, we distinguished the four genotypes with optimal criteria. Based on the quantitative criteria, we compared experimental results with vertex model simulations, which we explored in a wide range of differential tensions. The comparison enabled us to estimate different contribution of clone tensions and tension parameters to clone morphologies of distinct genotypes, noting that the force inference of individual cells over entire tissue is beyond the scope of the present paper. The present multivariate clone shape quantification could be extended to estimate genetic and non-genetic cell mechanics of heterogeneous populations (e.g., tumorigenic environment) in a non-invasive manner.

## Materials and methods

### *Drosophila* strains and genetics

We used *y w hs-flp; DE-Cad::GFP; Act*>*CD2*>*GAL4, UAS-DsRed* as the tester-stock genotype in our experiments. We crossed the tester stock with RNAi lines and raised the offspring at 25°C for 3 days. We then subjected the offspring to heat shock at 37°C for 40 min to induce somatic clones (Figure 1K). We subsequently kept the larvae at 25°C for 3 days before dissection. We used the following transgenic strains in our study: UAS-*trn* (Sakurai et al., 2007), UAS-*hbs* (Dworak et al., 2001), and UAS-ds-*Eph* (Vienna stock center, 4771). Hereafter, we refer to the *Drosophila* tester-stock clone as the wild-type.

### Immunohistochemistry

We hand dissected larvae to obtain wing imaginal discs, which we fixed in PBS with 4% formaldehyde for 40 min at room temperature. We washed the fixed samples three times with PBT (PBS with 0.1% triton) and mounted them on a glass slide.

### Imaging and image processing

We obtained images with a Leica SP8 confocal scanning microscope with a 40 × NA 1.30oil objective. We visualized adherens junctions with the localization of a GFP knock-in for DE-Cadherin (Huang et al., 2009) and used them for image segmentation. We manually selected the GFP signals derived from columnar cells of the wing pouch before making a z-stack projection. We projected the z-stack images by the maximum projection in Fiji (http://fiji.sc) and used them for further quantitative analysis. Average pixel size for each cell junction was 8.4 (Supplementary Figure S11).

### Clone shape quantification

We performed segmentation, cell tracking, and bond tracking (Figures 1P–S) using the Fiji plugin Tissue Analyzer (Aigouy et al., 2016). We projected the clones onto the segmented images and identified cells in the clones using Tissue Analyzer. We roughly estimated possible error rates by having 5 unexperienced individuals hand-correct a segmentation mask for one of the images we used in this study. We estimated the error rate in 4 ways as follows (Supplementary Figure S4); (1) the mean rate of hand-corrections made after auto-segmentation (0.84% of all cell junctions), (2) the mean rate of hand-corrections made by another person after the 1st round of hand-correction (0.28% of all cell junctions), (3) the mean rate of hand-correction made by 1st and 2nd round of hand-correction in total (1.12% of all cell junctions), and 4) the mean final discrepancy rate between 2 individuals (0.23%, max. 0.44%). We note that the correction rate highly depends on original image quality therefore the rate would be variable among images.

We quantified the clone shapes using multiple criteria. Circularity is a measure that calculates the ratio between the perimeter and the area of a clone and has been used to evaluate clone shapes (Figure 1C). We also used the following cell-based criteria: cell area (Figure 1D), cell edge length (Figure 1E), clone boundary angle (Figure 1F), and three types of cell mixing index (Figure 1G) [i.e., mutant (MT; Figure 1H), boundary of mutant (BDMT; Figure 1I), and boundary of wild-type (BDWT; Figure 1J)].

### Principal component analysis (PCA)

We performed PCA of the multi-dimensional criteria for clone shape using the R environment for statistical computing (R Development Core Team, 2015) with the “prcomp” function. We plotted the results using the “ggbiplot” function (R package version 0.55. http://github.com/vqv/ggbiplot). We applied PCA to both open and closed clones in the wing imaginal discs using the six criteria (Figures 1D–F,H–J) excluding circularity. We standardized the variables to have zero mean and unit variance before the analysis. Factor loadings (**Figure 3K**), which were given by the correlation coefficient between observed variables (criteria) and principal components (PCs), represent the contribution of criteria on PCs. The range of the value is −1.0 to 1.0. The value of −1.0 and 1.0 for the criteria indicates a perfect negative and positive correlation with the PCs, respectively.

### Cell vertex model

The cell vertex model quantitatively accounts for the packing geometry of normal epithelial cells and predicts the forces that act at cell–cell interfaces, where cell configurations are described as polygons whose vertices form tri-cellular junctions subjected to mechanical force (Honda, 1983; Farhadifar et al., 2007; Gibson et al., 2011). The model can reproduce stable force balance configurations of the adherens junctions network, which depend on mechanical parameters characterizing the cell bond tension and apical area contraction. Cells change their shape based on the force balance of cell packing. The model is represented by balance of three types of mechanical force exerted on a vertex (Farhadifar et al., 2007):

$$\begin{array}{r} \frac{d\vec{x_{i}}}{dt}=F_{area\;elastisity}+F_{tension}+F_{contractility}=-\frac{\partial E}{\partial \vec{x_{i}}} \end{array}$$

(1) $$\begin{array}{r} E=\frac{1}{2}\sum_{\alpha }{\left(a_{\alpha }-1\right)}^{2}+\gamma \sum_{<i,j>}l_{ij}+\frac{\eta }{2}\sum_{\alpha }{l_{\alpha }}^{2}, \end{array}$$

where *x*_*i*_ and *E* are the position vector of each vertex and the energy function. *F*_*area elasticity*_ denotes the area elasticity, which decreases as the area of cell α (*a*_α_) approaches the normalized preferred area of unity. The line tension (*F*_*tension*_) between vertices *i* and *j* (*l*_ij_) is provided by the cell–cell adhesion mediated by adhesion molecules and the contractility exerted by actomyosin. The contraction (*F*_*area elasticity*_) of the cell perimeter *l*_α_ is provided by actomyosin ring. We set the contractility parameter η = 0.04 and the line tension parameter γ = 0.12 as the control values to account for the cell packing geometry in *Drosophila* wild-type clones (Farhadifar et al., 2007). The line tension at the clone boundary (γ_b_) and at the inner clonal edges (γ_c_) can differ from γ. Each clone was generated from a single cell by dividing them to be 40 cells in total, which is close to the average number of cells in *Drosophila* wild-type clones. We performed 5 independent simulations for each parameter by changing the seed of the random number generator.

We integrated the vertex model numerically using the Euler method with free boundary conditions. To achieve a mechanical equilibrium of the tissue state, we calculated the position vector of each vertex after each step until the total velocity of all vertices dropped below a threshold of 1.0. The cell division time (cell cycle) *t* of each cell in the cell vertex model obeys a Gamma distribution with the following probability densities: $p\left(t\right)=t^{k-1}exp\left(-kt/\overset{®}{T}\right){\left(\overset{®}{T}/k\right)}^{-k}/\Gamma \left(k\right)$, where Γ*(k)* is the gamma function with *k* = 25.0 (Wartlick, 2011). The average and standard deviation are given by $\overset{®}{T}$ and $\overset{®}{T}/\sqrt{k}$, with $\overset{®}{T}=6$. Cells were divided when the residence times in the cell cycle became zero. Although the cell area decreases to half the original cell area after cell division, it increases as the cell achieves a mechanical equilibrium of energy [Equation (1)]. The mitotic cleavage-plane orientation obeys the long axis rule (Gibson et al., 2011; Bosveld et al., 2016b), where the plane passing through the shorter axis is defined by calculating the inertial tensor of each cell using the positions of the vertices (Fletcher et al., 2013). Cell intercalation (T1 transition) was incorporated when the edge length dropped below a threshold of 0.01. Apoptosis (T2 transition) was introduced into triangular cells (containing 3 vertices) whose area became below a threshold of 0.03 to replace them by a single vertex. In addition, cells which were squeezed and reduced their cell area below a threshold of 0.001 were eliminated by repeating the cell topological changes (T1 and T2 transition) even if the cells containing more than 3 vertices. The number of apoptotic events (during proliferation of a clone from a single cell to 40 cells) depends on the model parameters as seen in earlier studies on homogeneous tissue (Farhadifar et al., 2007). The apoptotic rate (number of apoptotic events/cell cycle) was 0 (γ_b_/γ = 0.5, γ_c_/γ = 1.8, **Figure 4Ai**), 1.5 (γ_b_/γ = 1.0, γ_c_/γ = 1.8, **Figure 4Aii**), 0 (γ_b_/γ = 0.5, γ_c_/γ = 1.0, **Figure 4Aiii**), 0 (γ_b_/γ = 1.0, γ_c_/γ = 1.0, **Figure 4Aiv**), 0.08 (γ_b_/γ = 1.6, γ_c_/γ = 1.0, **Figure 4Av**), 0 (γ_b_/γ = 1.0, γ_c_/γ = 0, **Figure 4Avi**), 0 (γ_b_/γ = 1.6, γ_c_/γ = 0, **Figure 4Avii**).

### Clone tension

Clone tension is given by the balance of line tension parameters [Equation (1)] at the three types of edges represented by γ, γ_b_, and γ_c_ (Bosveld et al., 2016a):

(2) $$\begin{array}{r} \hat{\sigma }=\frac{\partial E}{\partial L}=\gamma_{b}-\frac{\gamma +\gamma_{c}}{2}. \end{array}$$

It represents the energy cost *E* [Equation (1)] per unit length of changes in the clone boundary length *L* (Figure 1C) followed by cell intercalation. Because the differential of elastic energy ∂*E*/∂*L* should be negative to satisfy a stable equilibrium, the sign of the clone tension $\hat{\sigma }$ influences the evolution of the clone boundary length *L*. At negative $\hat{\sigma }$, boundary length change δ*L* should be positive to increase the contact between different cell populations, leading to a convoluted clone boundary. In contrast, at positive $\hat{\sigma }$, δ*L* should be negative to decrease the contact between different cell populations, leading to a smoothed clone boundary. We used dimensionless clone tension $\sigma =\hat{\sigma }/\gamma$ (Bosveld et al., 2016a) in this study.

### Projection of the simulation data onto the PCA space of the experimental data

To quantitatively compare the multivariate clone shape data from genetic experiments with the vertex model simulations in the PCA space constructed from the experimental data (Figure 1A), the simulation clones (See Section Cell Vertex Model for induction of clones) were quantified by an identical set of quantitative criteria with experiments (Figures 1D–F,H–J). Subsequently, we subtracted the clone shape dataset for each criterion in simulations from the mean of that in the four genotypes and divided the subtracted values by the standard deviation of that in the genotypes. We calculated the first PC scores (*Y*_1_) of the scaled simulation data (*X*_1_, *X*_2_, …., *X*_*n*_) by *Y*_1_ = *a*_11_*X*_1_ + *a*_12_*X*_2_ +…+ *a*_1*n*_*X*_*n*_, where *a*_11_, *a*_12_, …, *a*_1*n*_ are the weights (Supplementary Table S1) of four genotypes with *n* = 6 (number of criteria; Figures 1D–F,H–J). The weights are given by the first column of the PC matrix, which contains variable loadings whose values are returned by “rotation” in the prcomp function in R. The second PC scores are given in the same way by using the second column of the matrix. We projected the calculated first and second PC scores onto the experimental PCA space using the “ggplot2” function in R (Wickham, 2009). The PC scores in simulation were averaged over the clones categorized by the number of consisting cells (every 5 cells) since the clone size was varied due to fragmentation of a single clone caused by T1 transitions in a simulation.

### Estimation of the mechanical parameters of the genetic experiments in the PCA space

We determined the estimated mechanical parameters by simulation plots inside a confidence ellipse, which we drew by assuming that the experimental plots of each genotype followed the multivariate normal distribution. We selected the best representative parameters for each genotype by identifying the shortest Mahalanobis distance from the center of the confidence ellipse.

## Results

### Clone shape quantification

**Figure 2:**
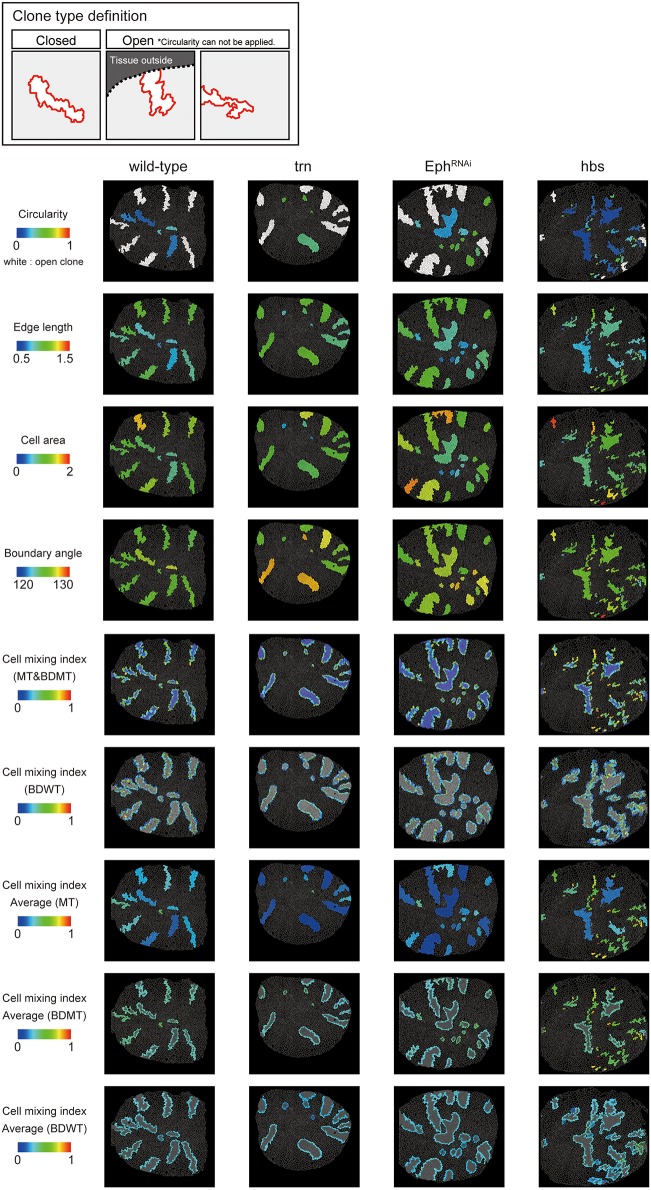
Clone shape quantifications in wing discs. Uppermost panels: Definition of clone type. “Closed” clone is completely enclosed by wild-type cells (left), while “Open” clone contains an invisible portion due to its location at the distal region of the tissue (middle) or image frame (right). Visualization of individual criteria (Figures 1C–J) for the examined clones of four genotypes (Figures 1L–O). For open clones, circularity was not measurable, so they were filled by a white color.

**Figure 3:**
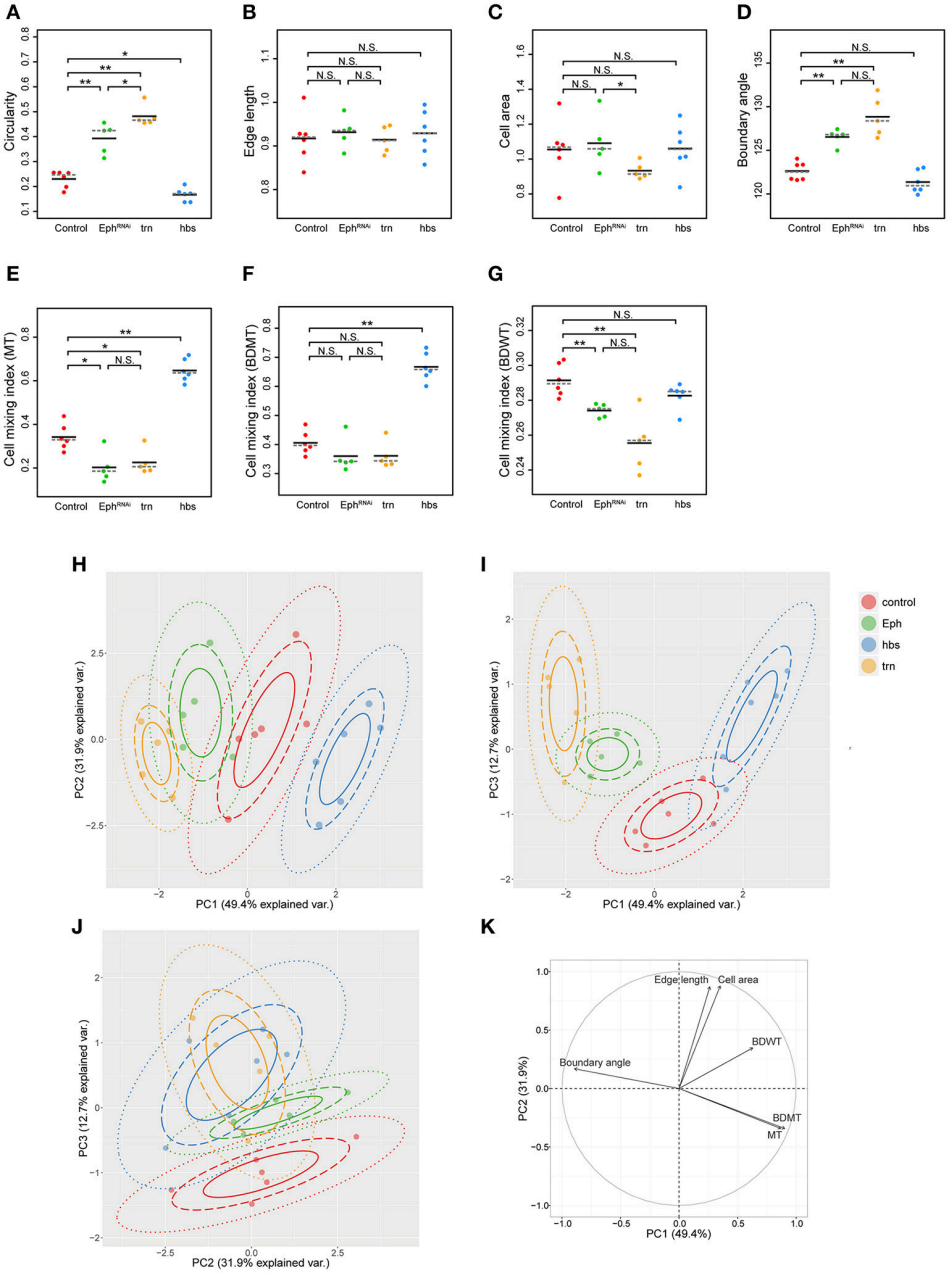
Principal component analysis of clone shape indicators for four genotypes. **(A–G)** Plots for circularity **(A)**, edge length **(B)**, cell area **(C)**, boundary angle **(D)** and cell mixing index of MT cells **(E)**, BDMT cells **(F)**, and BDWT cells **(G)** of clones of four genotypes including wild-type (red), *Eph* RNAi (green), *trn* overexpression (yellow), and *hbs* overexpression (blue). The plotted data were obtained from clones with averaging within a disc. Black solid lines and gray broken lines represent the average and median values, respectively. Wilcoxon rank sum test, *n* = 6 for control and *hbs* overexpression; *n* = 5 for *Eph* RNAi and *trn* overexpression. ^**^P < 0.01, ^*^P < 0.05 and *P* > 0.05 (N.S., Not Significant). **(H–J)** Results of principal component analysis using six criteria (edge length, cell area, boundary angle, and cell mixing index of MT cells, BDMT cells, and BDWT cells) are plotted for PC1 vs. PC2 **(H)**, PC1 vs. PC3 **(I)**, and PC2 vs. PC3 **(J)**. **(K)** Quantified contribution (factor loadings; Section Principal Component Analysis (PCA) for definition) of each criterion on PC1 and PC2. The arrow length represents the sum of the squared correlation coefficient of PC1 and PC2, noting that the sum of all PCs is equal to 1.

We adopted several geometrical indicators (Figures 1B–J and Supplementary Figures S1, S2) to quantitatively evaluate the shape of mosaic clones of distinct genotypes induced in the wing discs (Figures 1K–O). First, we calculated the circularity of each clone (Section Clone Shape Quantification; Figures 1C, 2), and Supplementary Figure S1). The average circularity of the *trn*-overexpression clones, which exhibited a round shape with a smooth border (Figure 1M), was significantly greater than that of the wild-type clones, as reported previously (Figure 3A; Milán et al., 2002). In the other genotype, *hbs*-overexpression clones, circularity was distinct from that of the wild-type clones (Figure 3A). The cells overexpressing *hbs*, however, scattered into many single-cell clones (Figure 1O and Supplementary Figure S3), so the circularity of the single-cell clones represented merely the shape of cells, and not that of clones. Moreover, circularity cannot be applied to open clones located at the edges of the images, because the perimeter length and area of those clones cannot be precisely determined (Figure 2, uppermost panels and white-colored clone in Circularity panels). Circularity can be applied only for clones with closed circumference and is therefore measurable only for those clones with a relatively small number of cells located in the middle of the tissue, and the entire clone should lie within the image frame (Figure 2, uppermost panels). In the *Drosophila* wing discs, which are frequently used for mosaic clone analysis, approximately half of the clones were open clones (46.4% for wild-type clones). Therefore, a cell-based analysis to quantify the clone shape was needed.

In order to quantify clone shape at the single-cell level, we precisely traced (segmented) the individual cell shapes and labeled the cells using Tissue Analyzer (Aigouy et al., 2016; Section Clone Shape Quantification; Figures 1P–S), which has a sufficiently low error rate of image segmentation (Supplementary Figure S4). For the segmented images, we measured the cell mixing index, which has been used to quantitatively evaluate how much a single cell shares junctions with a neighboring population (Umetsu et al., 2014a; Levayer et al., 2015) (Figures 1G–J, 2; and Supplementary Figure S1; MT, BDMT, and BDWT). Remarkably, the MT and BDMT of the *hbs*-overexpression clones were significantly higher than those of the wild-type clones (Figures 3E,F). In order to test whether the cell mixing index is sensitive enough to distinguish clone shapes with different smoothness, we compared *Eph*-RNAi clones (Figures 1N,R) with *trn*-overexpression clones (Figures 1M,Q); however, none of the indices (MT, BDMT, and BDWT) was able to distinguish between the *Eph*-RNAi clones and the *trn*-overexpression clones (Figures 3E–G). The boundary angle (Figure 1F) was also unable to separate the *Eph*-RNAi and *trn*-overexpression clones, although it could distinguish the *Eph*-RNAi or *trn*-overexpression clones from the wild-type clones (Figures 2, 3D). The edge length (Figure 1E) was not able to distinguish between any pair of genotypes (Figures 2, 3B). The cell area (Figure 1D) could uniquely distinguish the *Eph*-RNAi clones from the *trn*-overexpression clones but not from the wild-type clones (Figure 3C). In summary, there was no single cell-based criterion that could separate the four genotypes. The combinatory use of multiple criteria may provide a better resolution to distinguish the clone shapes of the genotypes, which could ultimately infer mechanical parameters of each genotype (Figure 1A).

### PCA separated sources of phenotypic heterogeneity

The combinatorial use of multivariate (6-dimensional) dataset increases information of data sets required for the complete separation, while the multi-dimensional information is too complex for us to intuitively extract some important criteria for the efficient separation. Therefore, multi-dimensional analysis generally has a trade-off between the merits and demerits. The principal component analysis (PCA) can optimize the trade-off: It can provide criteria to maximize the variance of the data and compresses multi-dimensional information into lower dimensions while retaining most of the original information. PCA has been extensively developed in the field of morphometrics of individual cells (Lacayo et al., 2007; Pincus and Theriot, 2007), organs (Iwata, 2002; Klingenberg, 2011), and individual bodies (Zelditch et al., 2012), but not of clones in multicellular tissue. We applied PCA to the data set of the six criteria for the four genotypes (*n* = 84 clones from six discs for wild-type, *n* = 61 clones from five discs for *Eph* RNAi, *n* = 53 clones from five discs for *trn* overexpression, *n* = 215 clones from six discs for *hbs* overexpression; Figures 3H–K and Supplementary Figures S5, S6). We averaged over the set of clones in each wing disc before applying PCA to analyze genetic variation separately from non-genetic variation. We found that more than 80% of the information was compressed into only two principal components (PC1 = 49.4%, PC2 = 31.9%, PC3 = 12.7%; Figures 3H–J). Each genotype was separated mainly in PC1 without overlap (Figure 3H), while the *Eph*-RNAi and *trn*-overexpression clones were also efficiently separated in PC2 and PC3 (Figures 3H–J). The major criteria with higher factor loading on PC1 [>0.85; See Section Principal Component Analysis (PCA) for the definition of factor loading] were MT, BDMT, and boundary angle, while some other criteria such as, cell area and edge length showed a high contribution on PC2, and BDWT contributed relatively on PC1 and PC3 (Figure 3K and Supplementary Figure S5). Both the separation of genotypes mainly in PC1 and the contribution of each criterion on PC1–PC2 were nearly the same “with averaging” or “without averaging” within the discs (Figures 3H–K and Supplementary Figure S6).

Note that PCA without averaging within the discs showed that the variation within a genotype was mainly distributed in PC2, while each genotype was separated in parallel with PC1 (Supplementary Figure S6A). Since the variation occurred in an identical combination of clone (e.g., *Eph* RNAi) and non-clone genotypes (wild-type), hereafter we call it “non-genetic” heterogeneity (Supplementary Figures S1, S7A, middle panels). Interestingly, PC2 had a positive correlation with the distance from the center of the disc (Supplementary Figure S7B), indicating that the non-genetic heterogeneity in PC2 encoded the positional information of each clone. That might be caused by spatial gradients of the expression of several genes in the *Drosophila* wing pouch (e.g., *spalt, optomotor-blind, Distalless*, and *vestigial*; Milán et al., 2002; Affolter and Basler, 2007; Swarup and Verheyen, 2012). Therefore, genetic as well as non-genetic heterogeneities were efficiently segregated in lower dimensions in the PC space, allowing us to estimate the mechanical basis of the genetic heterogeneity using PCA.

### Vertex model simulations with differential line tension

**Figure 4:**
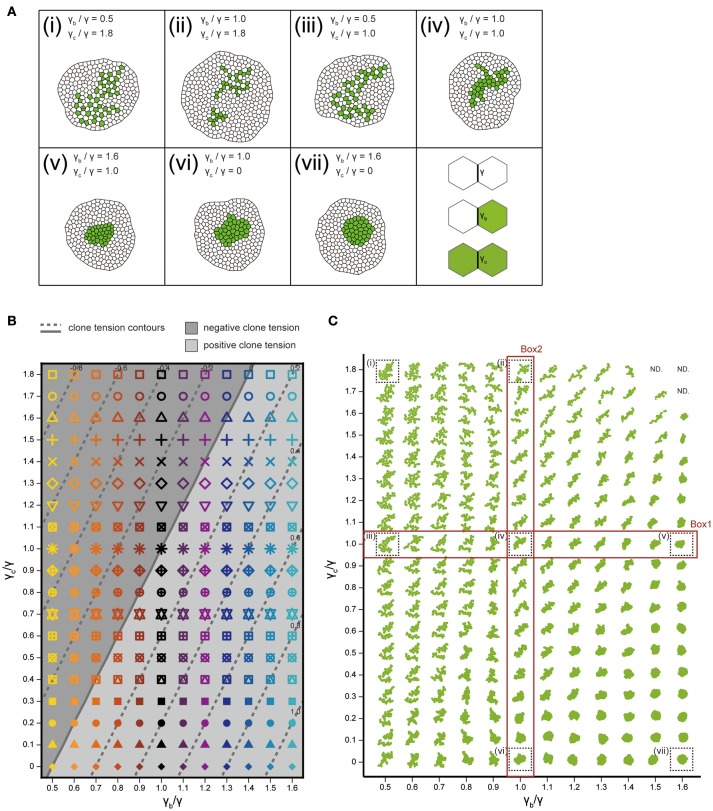
Cell vertex model simulations. **(A)** Right bottom: The classification of line tension parameters: γ [default tension at edges between non-clonal cells (white)], γ_b_ (clone boundary tension at edges on the clone boundary), and γ_c_ [inner clonal tension at edges between clonal cells (green)]. **(i–vii)** Snapshots of simulated clones when the total number of green cells is 40. **(B)** Parameter space of normalized line tension (γ_b_/γ and γ_c_/γ) with contour lines of clone tension σ (See Section Clone Tension for definition). Each symbol denotes different combinations of parameters (γ_b_/γ, γ_c_/γ). **(C)** Representative form of the clones for all simulation parameters in **(B)**, noting that the model did not work at the three parameter points indicated by “ND.” Clones enclosed within black dashed boxes are the same as those in **(i–vii)**.

We analyzed how the mechanical cell–cell interactions contribute to the characteristic clone shapes (Figures 1L–S) by comparing *Drosophila* experiments to computer simulations (Figure 1A). We utilized the cell vertex model, which quantitatively accounts for the packing geometry of epithelial cells [Section Cell Vertex Model, Equation (1)]. Clone shape has been shown to be modulated mainly by the combination of line tension parameters at three types of edges: γ [default tension in Equation (1)], γ_b_ (clone boundary tension), and γ_c_ (inner clonal tension; Figure 4A, right bottom panel for the classification of edges) (Graner, 1993; Graner and Sawada, 1993; Brodland, 2002). Therefore, as a pilot study for the multivariate inference of mechanical parameters for each genotype, we presupposed that γ_b_ and γ_c_ relative to γ are the main causes of clone formation, although other parameters such as, the rate of cell proliferation might potentially influence the clone boundary shape (see Supplementary Figure S3 for detailed data on the numbers of cells and clones). We numerically explored the role of the different combinations of line tension parameters γ_b_ and γ_c_ in the formation of clones. As the tension parameter for clone boundary γ_b_ increased relative to the control tension parameter γ, cells were sorted out so that the clone boundary became smoothly rounded (Box1 in Figure 4C), as seen previously (Landsberg et al., 2009; Monier et al., 2010; Aliee et al., 2012; Rudolf et al., 2015; Bosveld et al., 2016a). We observed a similar tendency to generate smooth clones by decreasing the inner clonal tension parameter γ_c_ relative to γ, which is equivalent to increasing adhesion between the inner clonal cells (Box2 in Figure 4C). Conversely, cell mixing occurred to form convoluted clones as γ_b_ decreased or γ_c_ increased relative to γ (Figure 4C). Those results indicate the differences between the default tension (γ) and the one at the clone boundary and inside the clone (γ_b_ and γ_c_) reflect cell sorting and cell mixing. As a measure to distinguish between cell sorting and mixing, we examined the dimensionless clone tension, which is represented by γ, γ_b_, and γ_c_ (σ in Section Clone Tension) (Bosveld et al., 2016a). We confirmed that negative and positive values of the clone tension (Figure 4B, gray and light-gray, respectively) mainly distinguished convoluted and rounded clone morphologies, respectively (Figure 4C).

### Inference of tension parameters by comparison between experiments and simulations in the PC space

**Figure 5:**
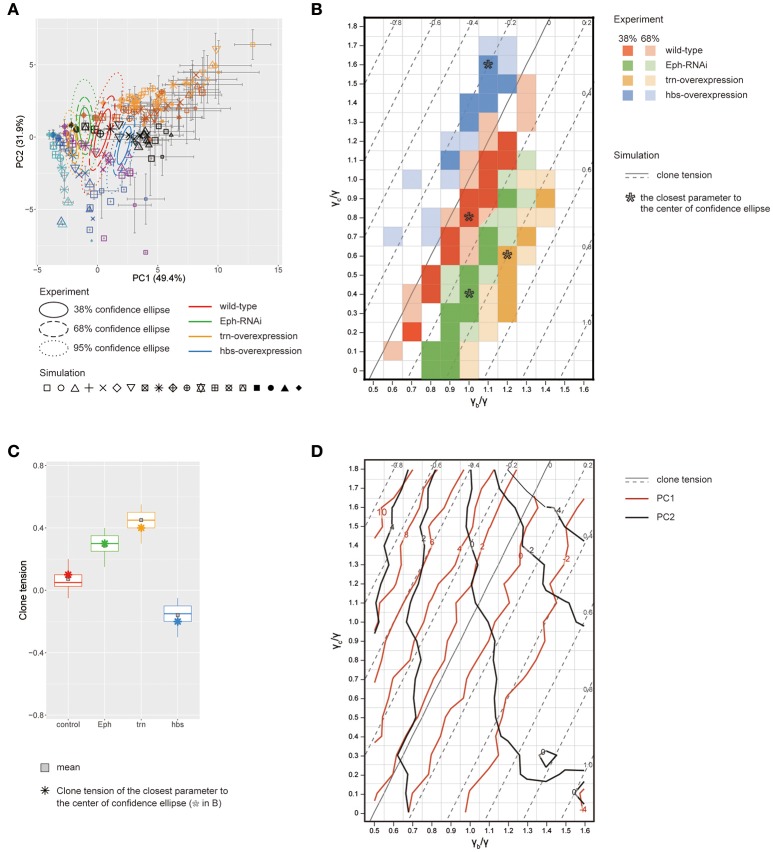
Inference of tension parameter and clone tension of four genotypes. **(A)** Projection of simulated clones onto the PC space of *Drosophila* experiments (See Section Projection of the Simulation Data onto the PCA Space of the Experimental Data for Projection; Symbols denote parameter values given in Figure 4B). A limited set of simulation points are plotted for visibility (see Supplementary Figure S12 for the projection of the entire simulated dataset). The experimental confidence ellipses were identical to those in Figure 3H. The averaged PC scores were plotted with symbol size proportional to the number of cells within a clone and error bar representing standard deviation. **(B)** Estimated mechanical parameters of four genotypes. Mechanical parameters within the 38 and 68% confidence ellipses in **(A)** are shown by dark and light colors, respectively. Pentagon asterisks (^*^) mark the best representative parameters, which are the closest to the center of each confidence ellipse. **(C)** Estimated clone tension of four genotypes calculated by the estimated mechanical parameters within the 68% confidence ellipse [dark and light colors in **(B)**]. The upper/lower hinge and thick middle line represent the 25th/75th and 50th percentiles, respectively. Gray squares show the averaged clone tension. Octagonal asterisks are the clone tension of the best representative parameters [pentagonal asterisks in **(B)**]. **(D)** Projection of the contour lines of PC1 and PC2 scores onto the parameter space of vertex model simulations.

We quantitatively compared the clone shapes generated by vertex model simulations using the control parameters (γ = γ_b_ = γ_c_, Figure 4Aiv) to those of *Drosophila* wild-type clones by projecting the simulated clones onto the PC1 and PC2 space of the experimental data (Figure 3H). The simulated control clones (Figure 5A, black octagonal asterisk at PC1 ~ 1.07, PC2 ~ 0.56) were consistently similar to the wild-type clones in the PC space (Figure 5A, red confidence ellipse). We inferred the wild-type tension parameters from the simulations plotted inside the 68% confidence ellipse of the wild-type clones (Section Estimation of the Mechanical Parameters of the Genetic Experiments in the PCA Space). The estimated parameters of the wild-type clones were distributed around the control parameters of the simulation (Figure 5B, red), where the clone tension was nearly zero (Figure 5B, solid line, and Figure 5C, red), indicating that the vertex model could closely coordinate with the PCA of static tissue images to non-invasively estimate the mechanical parameters of the wild-type clone based only on the clone morphology.

Likewise, when we simultaneously projected simulation data from a wide range of tension parameter sets (all marks in Figure 4B) onto the PC space, we found that the two-dimensionality of the parameter space of the line tension (γ_c_/γ and γ_b_/γ; Figure 4B) was maintained in the PC1-PC2 space (Figure 5A), indicating a quantitative link between the tension parameters at the cell–cell interface and the clone boundary geometry of heterogeneous populations. Therefore, following the methods applied to the wild-type clone, we estimated the mechanical parameters of the other three genotypes (*Eph* RNAi, *trn* overexpression, and *hbs* overexpression). We found that the estimated regions of the mechanical parameters for each genotype (plotted inside the 68% confidence ellipses in Figure 5A) were distributed in parallel to the contour lines of the clone tension (Figure 5B). For the *Eph*-RNAi and *trn*-overexpression clones, which had a rounded morphology (Figures 1M,N), the tension of both clones was higher than that of the wild-type clone (Figure 5C). The *hbs*-overexpression clones had a negative value for clone tension (Figure 5C), which was consistent with their convoluted morphology (Figure 1O). The ascending order of clone tension (*hbs* overexpression < wild-type < *Eph* RNAi < *trn* overexpression; Figure 5C) perfectly agreed with that of PC1 (Figure 3H), indicating that the value of PC1 reflects the difference in clone tension. To more directly relate the clone tension to the PCA, we projected the PC scores onto the parameter space of the vertex model simulations (Figure 5D). The contour of the PC1 scores was almost parallel to that of the clone tension (Figure 5D, red solid line), whereas that of the PC2 scores was rather perpendicular to that of the clone tension (Figure 5D, dark gray solid line), indicating that the clone tension was the mechanical basis of PC1.

**Figure 6:**
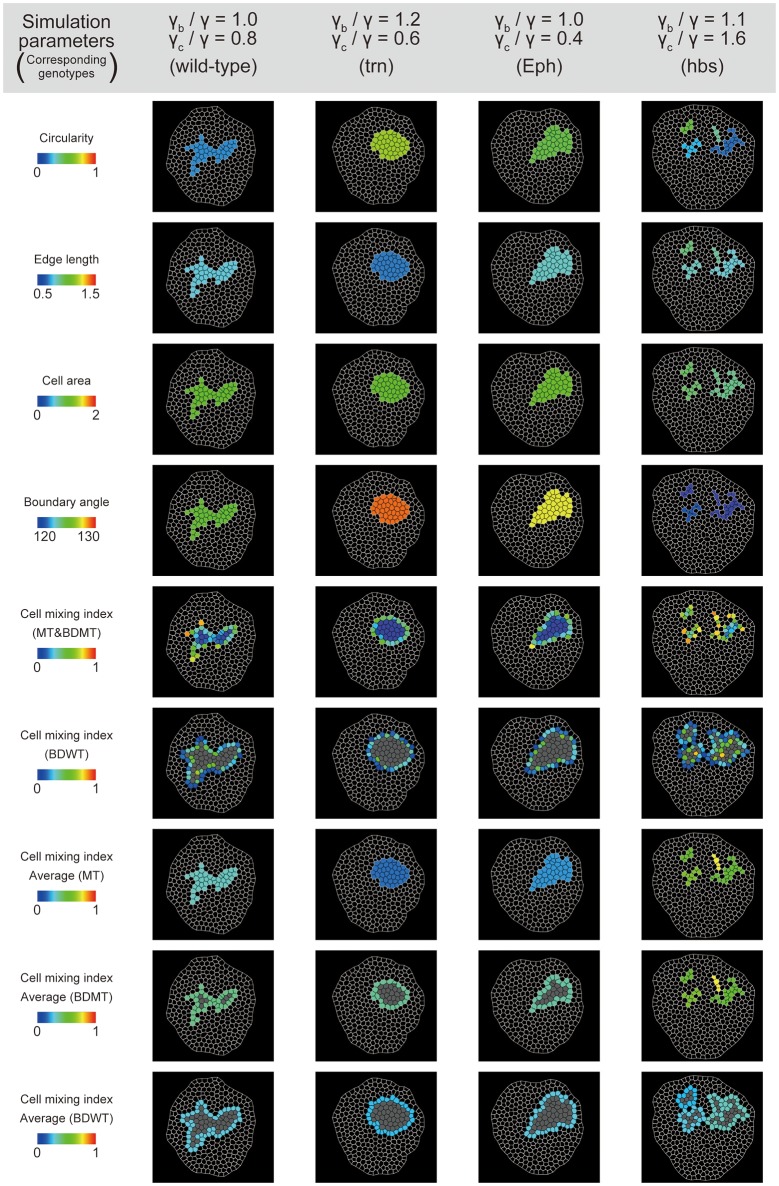
Simulation clones corresponding to the experimental clones. Visualization of individual criteria for the four simulated clones, which correspond to the best representative combination of line tension parameters for the four genotypes (pentagon asterisks in Figure 5B).

To better estimate the tension parameters γ_b_ and γ_c_, we defined the simulation plots closest to the centers of the confidence ellipses (Figure 5A) as the best representative combinations of line tension parameters for each genotype (pentagonal asterisks in Figures 5B, 6). We confirmed that the representative parameters γ_b_ and γ_c_ in simulations quantitatively reproduced experiments of each genotype according to all clone shape criteria used in PCA (Supplementary Figures S8A, S9A, left most panels). The representative parameters indicated that the cause of the higher clone tension was different between the *Eph*-RNAi clones and the *trn*-overexpression clones. The difference was mainly caused by dominantly reduced γ_c_ relative to γ for the *Eph*-RNAi genotype and both reduced γ_c_ and increased γ_b_ relative to γ for the *trn*-overexpression genotype (Figure 5B). The lower clone tension of the *hbs*-overexpression clones was caused by dominantly increased γ_c_ relative to γ (pentagonal asterisk in Figure 5B).

At a constant clone tension for each genotype estimated by PC1, PC2 is sensitive to the combination of γ_b_ and γ_c_ (e.g., −4 ≦ PC2 ≦ 2 at clone tension = −0.2 in Figure 5D), so that the dominant change of γ_b_ or γ_c_ relative to γ was determined according to PC2 value of each genotype. The underlying mechanics how PC2 distinguished the dominance can be understood from the mechanical equilibrium of vertex model; edge length at clone boundary and cell area inside clones, which had higher contribution to PC2 (Figure 3K), become smaller as line tension strength γ_b_ and γ_c_ at constant clone tension increase in simulations (Supplementary Figure S10). Thus, the mutual projection of the mechanical parameter space and the PC space could efficiently and quantitatively estimate the distinct mechanics of the various clone morphologies.

## Discussion

### Multivariate analysis of clone shapes

We first developed a PCA of clone shapes in heterogeneous cell populations, which efficiently segregated clones of each genotype in the PC space. No single cell-based criterion of clone shape was able to perfectly segregate all four genotypes (Figure 3). In addition, because most of the conventional ways to quantify clone shape used in previous studies can only apply to closed clones (e.g., circularity, Figure 1C), much information regarding open clones (e.g., white clones in Figure 2) was previously lost during the quantification process. Open clones are frequently observed, however, in tissue specimens. The cell-based criteria that we used (Figures 1D–J) can apply to not only closed clones but also open clones, so we can use them widely and independently on the clone shapes and its location in the tissue. Furthermore, the average pixel size for each cell junction was only 8.4 (Supplementary Figure S11), suggesting the relatively low resolution imaging data is sufficient to distinguish clones of different genotypes with a minimal manual correction (1.12%, Section Clone Shape Quantification and Supplementary Figure S4). Therefore, our cell-based quantification provides a robust method for the clone shape quantification.

Both genetic and non-genetic heterogeneities influence tumorigenesis (Cortina et al., 2007; Porazinski et al., 2016), cell competition, and other events. Elimination of loser cells at the clone boundary during cell competition has been shown to be driven by genetic heterogeneity (Vincent et al., 2013; Amoyel and Bach, 2014; Morata and Ballesteros-Arias, 2015), while not all of loser cells are eliminated indicating the non-genetic heterogeneity due to local cell–cell contacts and gene expression levels in tissues might affect the elimination [e.g., Figure 1 in Levayer et al. (2015); (Froldi et al., 2010; Chen et al., 2012; Kajita et al., 2014)]. The PCA applied to individual clones efficiently separated the genetic heterogeneity mainly in PC1, while the non-genetic heterogeneity was distributed in PC2 (Supplementary Figures S6, S7), indicating that PC1 and PC2 encode genetic and non-genetic heterogeneities, respectively. That in turn indicates that the criteria with high contributions on PC1 (BDMT, MT, and boundary angle; Supplementary Figure S6D) and PC2 (BDWT, cell area, and edge length; Supplementary Figure S6D) optimally indicate the genetic and non-genetic heterogeneity, respectively, for the four genotypes in our study. The determination of whether those criteria commonly distinguish between genetic and non-genetic heterogeneity in other genotypes in the future will give clues as to how to separately evaluate genetic and non-genetic contributions to physiological functions of heterogeneous tissue.

### Non-invasive estimation of cell mechanics

We non-invasively estimated the mechanical parameters (i.e., clone tension and line tensions) of each genotype by integrating in a PC space the results of vertex model simulations and the quantified clone shapes in static images of cells (Figure 5A and Supplementary Figure S12). As a case study for rounded clones, we revealed that *trn*-overexpression clones have greater clone tension than *Eph*-RNAi clones (Figure 5C). Despite the patterned expression and the known, spatially graded function of *trn* in the wing pouch (Milán et al., 2002), our method successfully captured the characteristic features of *trn* overexpression, which are significantly distinct from those of *Eph* knockdown (Figures 3H–K). That suggests that our method can be applied to the coarse classification of clones with distinct genotypes even in the presence of non-genetic variation derived from a spatial gradient.

We also confirmed that the clone tension of the wild-type was nearly zero (Figure 5C). In addition, the range of clone tension for the other genotypes was also close to that measured previously (0 < σ < 0.6) (Bosveld et al., 2016a), despite the fact that we used different genotypes (Figure 5C). In addition, difference of the estimated clone tension (asterisk of Figure 5C) among genotypes was larger than the variation within each genotype (box height of each plot in Figure 5C), which was also confirmed by the estimation precision of clone tension [±0.15 (wild-type), under ±0.1 (*Eph*), ±0.1 (*hbs*), ±0.2 (*trn*) calculated from estimation precision of γ_b_ and γ_c_ in Supplementary Figures S8, S9 using Equation (2)]. The results indicate that our estimated values were roughly correct and that our method works well to estimate the mechanics. Moreover, we first showed *in vivo* that negative clone tension causes a convoluted morphology by inducing *hbs*-overexpression clones (Figure 5C).

Our study revealed the cell mechanics, relative contributions of γ_b_ or γ_c_ to the increase or decrease in clone tension. By examining the underlying cell mechanics, we found that the relative contributions of the line tension parameters γ_b_ and γ_c_ were different among genotypes, even when the sign of the clone tension was the same. Estimation precision of γ_b_/γ for each genotype was evaluated by a limit to how much shift of γ_b_/γ from the best representative (pentagonal asterisks in Figure 5B) simulation could reproduce experimental data; It was at most ±0.2 [under ±0.1 (*Eph, hbs*), ±0.1 (wild-type), and ±0.2 (*trn*)] (Supplementary Figure S8), whereas estimation precision of γ_c_/γ was at most ±0.3 [±0.1 (*Eph*), ±0.2 (*hbs, trn*), and ±0.3 (wild-type)] (Supplementary Figure S9). The estimated parameter variation of γ_b_/γ and γ_c_/γ for each genotype (68% confidence, dark and light colors in Figure 5B) was consistently smaller than difference of the best representative parameters between genotypes (pentagonal asterisks in Figure 5B). The *Eph*-RNAi clone had decreased bulk line tension, whereas the *trn*-overexpression clone had both increased boundary tension γ_b_ and decreased γ_c_ (pentagonal asterisks in Figure 5B), resulting in positive clone tension. The *hbs*-overexpression clone, which showed cell mixing due to negative clone tension, mainly had increased γ_c_ (pentagonal asterisk in Figure 5B). Cell mixing caused by increased γ_c_ was reported previously in *myc*-induced cell competition (Levayer et al., 2015). Our inference on the dominance of γ_b_ or γ_c_ for each genotype should be verified by estimation of the mechanical tension by physical perturbation in the future (e.g., laser cutting of the three types of edges in Figure 4A, right bottom panel).

### Molecular mechanisms of cell sorting and mixing mechanics

The present estimation provides mechanical insights of the previously reported molecular functions in cell sorting (Figure 5B): *Drosophila trn* and its paralog *capricious* (*caps*) encode leucine rich repeat containing transmembrane proteins that have a function in controlling cell affinity and the regulation of cell communication for cell survival (Milán et al., 2001, 2002; Sakurai et al., 2007). The mammalian homolog, Lrrn1 is required for the formation of the midbrain-hindbrain boundary by regulating cell affinity (Tossell et al., 2011). Eph kinases comprise a large protein family of receptor protein tyrosine kinases. Eph receptors are activated by binding to ephrins, their membrane-anchored ligands, to transduce signals that play diverse roles in axon guidance, neural crest-cell migration, and boundary formation of rhombomeres through their function in repulsive cell–cell interactions (Sela-Donenfeld and Wilkinson, 2005; Fagotto et al., 2014). In addition, mammalian EphB signaling plays a tumor-suppressive role by compartmentalizing cancer cells, potentially through cell-repulsive activity by means of actin cytoskeleton reorganization (Cortina et al., 2007). Our result showing decreased line tension at homotypic cell junctions (γ_c_) within the *Eph*-RNAi clones (Figure 5B) is consistent with the previously proposed repulsive functions of Eph receptors. Therefore, our method was consistent with the previously reported cell functions. Given the common and uncommon biological functions between *trn* and *Eph*, it would be interesting to investigate how the difference in cell mechanics between the *trn*-overexpression clone and *Eph*-RNAi clone contributes to biological functions of those genes.

Our analysis showed that the overexpression of *hbs* alone can result in negative clone tension (Figure 5C), which leads to convoluted clonal morphology and cell scattering. The main contribution to the negative clone tension was the increase of γ_c_ (Figure 5B), which would be a reflection of either an increase in cortical contractility or a decrease in adhesion at homotypic cell junctions within the clone [Equation (2)]. That suggests that the scattered morphology of the *hbs-*overexpression clones was derived from increased cell bond tension at the *hbs*–*hbs* cell interface, which is independent of the interaction between heterotypic cell junctions at the wild-type–*hbs* interface. The mechanical basis of *hbs* function has been poorly understood, except for its heterophilic interaction with the other nephrin family proteins Roughest and Kirre. Our result therefore provides a new mechanical insight into the cell mixing mechanisms mediated by those proteins.

### Future problems

In addition to line tension parameters, the clone boundary shape could be affected by inter-clonal differences in other parameters such as, the cell proliferation rate, apoptosis rate, and cell division orientation, which we will incorporate into the vertex model in the future. For example, the proliferation rate appeared to be increased more often in *Eph*-RNAi cells than in wild-type cells (Supplementary Figure S3B). Differential proliferation rates induced by such mutants are known to cause not only clone boundary smoothing [e.g., *Ras*^V12^-expressing (constitutively active form) cells (Prober and Edgar, 2000) and *tkv*^Q253D^-expressing (activated form of Dpp receptor) cells (Nellen et al., 1996)] but also the mechanical elongation of slower-dividing cells at the clone boundary and the compaction of faster-dividing cells [e.g., *Hippo* mutant clones in wild-type tissue (LeGoff et al., 2013; Mao et al., 2013; Pan et al., 2016)], so that cell shape anisotropy, which is a new criterion for cell elongation (LeGoff et al., 2013; Mao et al., 2013), as well as BDWT, clonal cell area, and other parameters might be reliable criteria for differential proliferation. The regulation of cell division orientation depends on the pathway of planar cell polarity, morphogen gradient, and mechanical cell stretching (Gillies and Cabernard, 2011; di Pietro et al., 2016; Stooke-Vaughan et al., 2017). Specifically, misoriented or directionally oriented cell division inside or at the periphery of the clone results in a rounded or convoluted shape of the clone boundary (Li et al., 2009; Mao et al., 2011; Kale et al., 2016). Taken together, the incorporation of additional criteria is required (e.g., cell shape anisotropy) in order to precisely infer the most responsive parameters of genotypes.

According to the increasing dimensions of the parameter space, parameter estimation should be performed in the higher dimensions of the PC space. While we used the PC1-PC2 space containing 80% of the information for the current estimation (Figure 5A), PC3 contained about 10% of the information, which segregated *Eph*-RNAi clones from *trn*-overexpression clones (Figures 3I,J). Additionally, we could narrow down the estimated parameter region by combining our current analysis with other analyses, such as, a distribution of several criteria as in the parameter estimation of homogeneous epithelial tissue (Farhadifar et al., 2007; Aegerter-Wilmsen et al., 2010). For example, *hbs*-overexpression clones showed an anomalous distribution of cell numbers within clones due to an increasing fraction of clones with smaller numbers of cells (Supplementary Figures S3A,C), while wild-type clones show coherent morphologies (Figure 1L) due to the remarkable lack of cell rearrangement in imaginal discs (Gibson et al., 2006). That indicates that the combinatorial use of the clone size distribution (Supplementary Figure S13B) with PCA could estimate the parameters for *hbs*-overexpression clones in a narrower range (Supplementary Figure S13A, parameters covered by both blue and gray oblique lines). Future studies should also clarify the limits and applicability of the multivariate inference of cell mechanics by exploring more complex systems as well as a wider variety of genotypes.

The pipeline based on cell shape quantification developed in this study may be extended to an image-based cancer diagnosis. High-throughput microscopy image-based cancer prognosis has been developed and is expected to provide useful prognostic information for precision medicine (Yu et al., 2016). In addition to the potential contribution to such a classification of the cancer subtype or grade, our method has a potential to provide the mechanistic understanding of tumor development. Elucidation of mechanistic ground of tumor morphology may help cancer treatment planning. Moreover, manipulation of the clone tension at the interface between normal cells and cancer cells (γ_b_) as well as of that between cancer cells (γ_c_) can be an alternative clinical approach to suppress cancer progression either by limiting tumor invasion or by promoting cell competition.

## Author contributions

Conceived and designed the experiments: AT, DU, EK, and KF. Performed genetic experiments: DU. Performed statistical analysis and computer simulations: AT. Analyzed the data: AT, DU, and KF. Wrote the paper: AT, DU, and KF.

### Conflict of interest statement

The authors declare that the research was conducted in the absence of any commercial or financial relationships that could be construed as a potential conflict of interest.
