# Supplementary material for: Inference of Cell Mechanics in Heterogeneous Epithelial Tissue Based on Multivariate Clone Shape Quantification
Source: Front Cell Dev Biol. 2017 Aug 3;5:68. doi: 10.3389/fcell.2017.00068 (PMC5540905; doi:10.3389/fcell.2017.00068)
Supplement: Supplementary file 1 [file DataSheet1.pdf]

Supplementary Material

Inference of cell mechanics in heterogeneous epithelial tissue based on multivariate clone shape evaluation

[#]Alice Tsuboi[1], [#,]*Daiki Umetsu[2], Erina Kuranaga[2], *Koichi Fujimoto[1]
[1]Department of Biological Sciences, Osaka University, Toyonaka, Japan
[2]Graduate School of Life Sciences, Tohoku University, Sendai, Japan
[#]Equal contribution
*** Correspondence:**
Daiki Umetsu
umetsu@tohoku.ac.jp
Koichi Fujimoto
fujimoto@bio.sci.osaka-u.ac.jp

Supplementary Figures 1–13

Supplementary Table 1

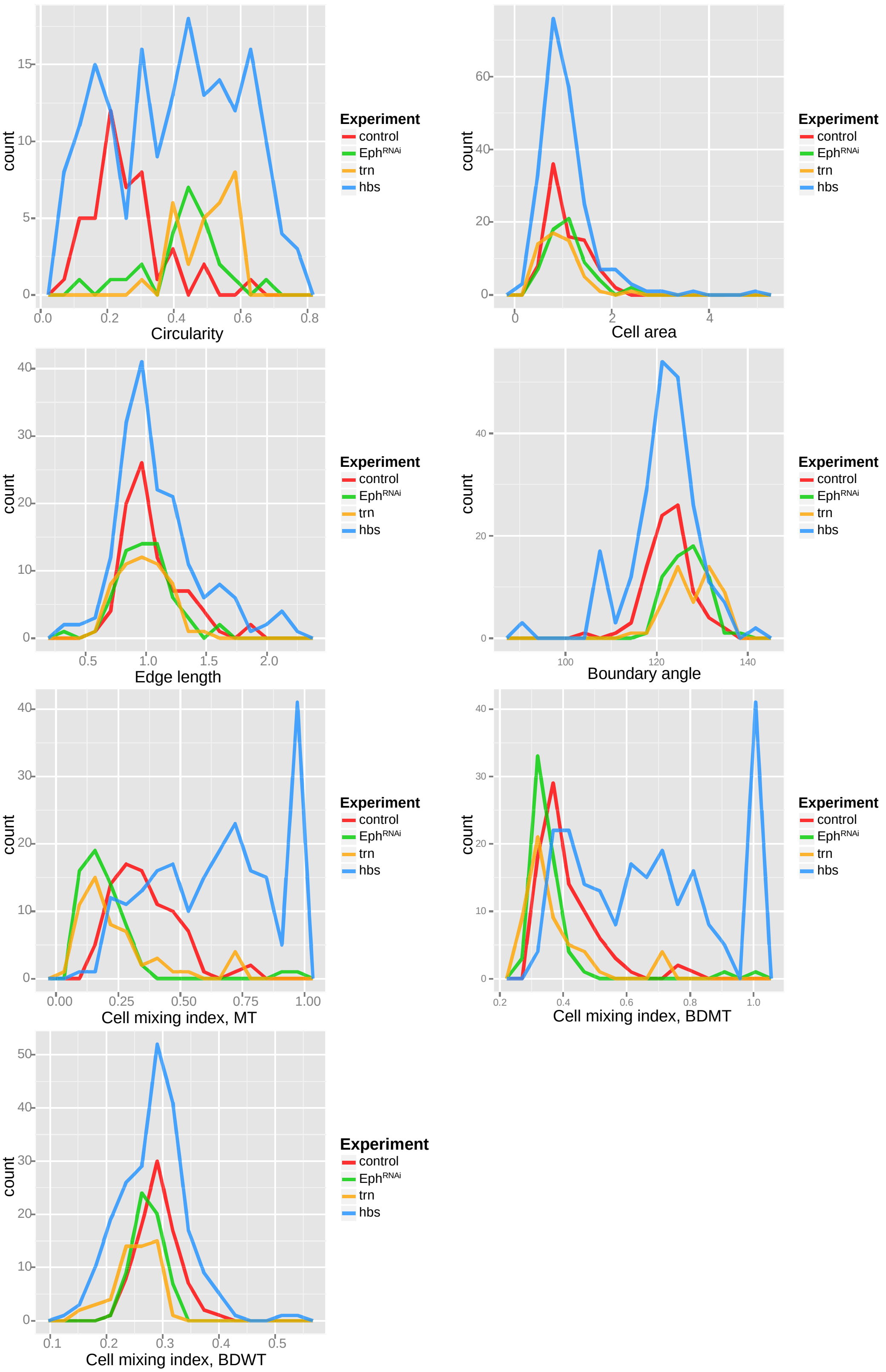

Supplementary Figure 1.

Histograms of individual criteria for the examined clones of the four genotypes shown in Figure 3A–G.

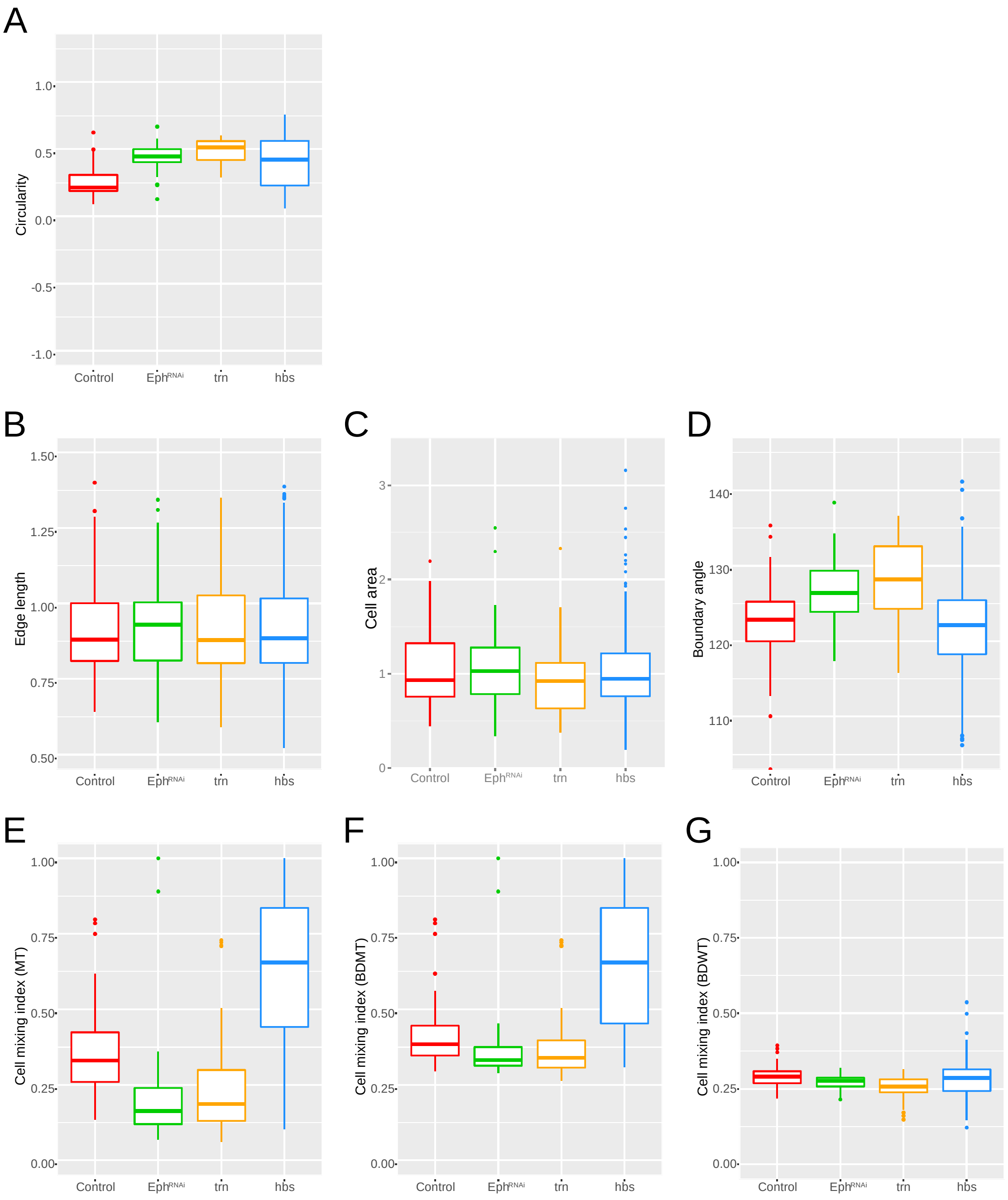

Supplementary Figure 2

(A-G) Box plots of circularity **(A)**, edge length **(B)**, cell area **(C)**, boundary angle **(D)**, and cell mixing index of MT cells **(E)**, BDMT cells **(F)**, and BDWT cells **(G)** for the examined clones of four genotypes: control (red), *Eph* RNAi (green), *trn* overexpression (yellow), and *hbs* overexpression (blue). Plotted data were obtained from individual clones without averaging within a disc (see Figure 3 for "with averaging"). We used only closed clones for circularity and both open and closed clones for the other criteria. The upper/lower hinge and thick middle line represent the 25th/75th and 50th percentiles, respectively, whereas colored dots denote outliers. We note that a few outliers with extremely high values of cell mixing index (MT, BDMT) in Eph denote clones containing only one or two cells inside of them.

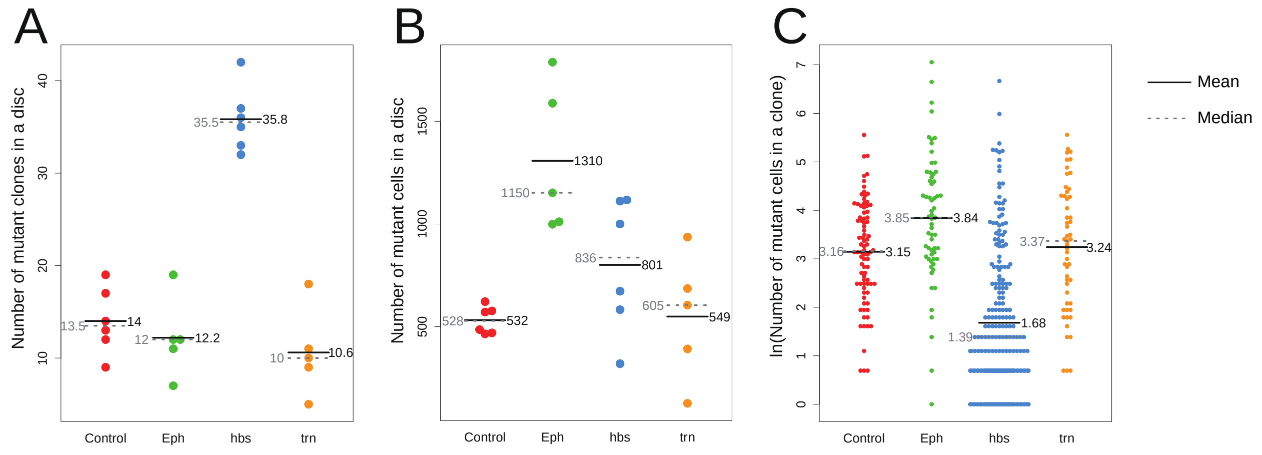

Supplementary Figure 3.

Each dot represents the number of clones **(A)** or cells **(B)** within a disc or the number of cells within a clone **(C)**. Black solid lines and grey broken lines represent the average and median values, respectively.

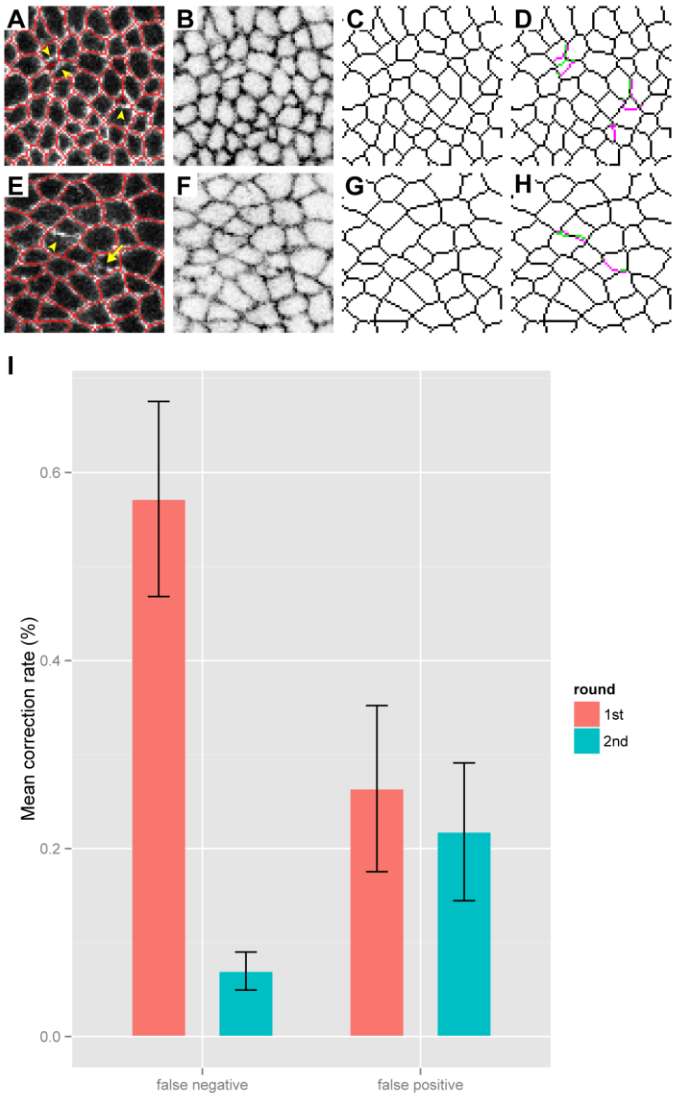

Supplementary Figure 4.

Estimation of segmentation error. **(A) and (E)** Overlay of a segmentation mask on its original image after auto-segmentation. Two magnified regions of an image sample from the *Eph* RNAi experiment. **(B)** Only the original image in (A) is shown. **(C)** Only the segmentation mask in (A) is shown. **(D)** Overlay of a hand-corrected segmentation mask on the auto-segmentation mask. Removed bonds from the auto-segmentation mask are shown in green and newly added bonds are shown in magenta. **(F)** Only the original image in (E) is shown. **(G)** Only the segmentation mask in (E) is shown. **(H)** Overlay of hand-corrected masks by different individuals. Note that while one bond was corrected in both masks (magenta and green bonds are aligned side by side) the other one exhibited discrepancy. Only one person considered it as a bond (magenta bond). Arrowheads point unrecognized cell bonds (false negative) and an arrow indicates a divisive cell bond. **(I)** Mean correction rate made by 5 individuals. Hand-corrections were performed 2 rounds by different persons for each round. The mean rate of hand-correction made by 1st and 2nd round in total was 1.12 % of all cell junctions (The sum of mean false negative and positive in 1st and 2nd round of hand-correction). Mean final discrepancy rate between 2 individuals was 0.23 % (max. 0.44 %).

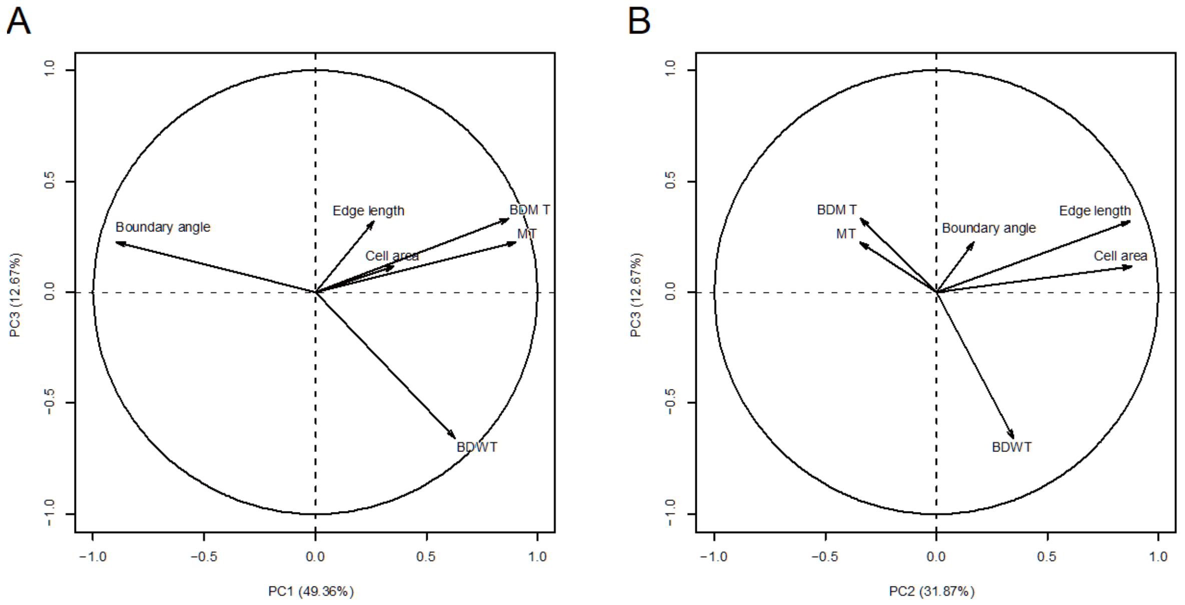

Supplementary Figure 5.
(A–B) Factor loadings of each criterion on PC1, PC2, and PC3 corresponding to the PCA with averaging within discs shown in Figure 3H-J.

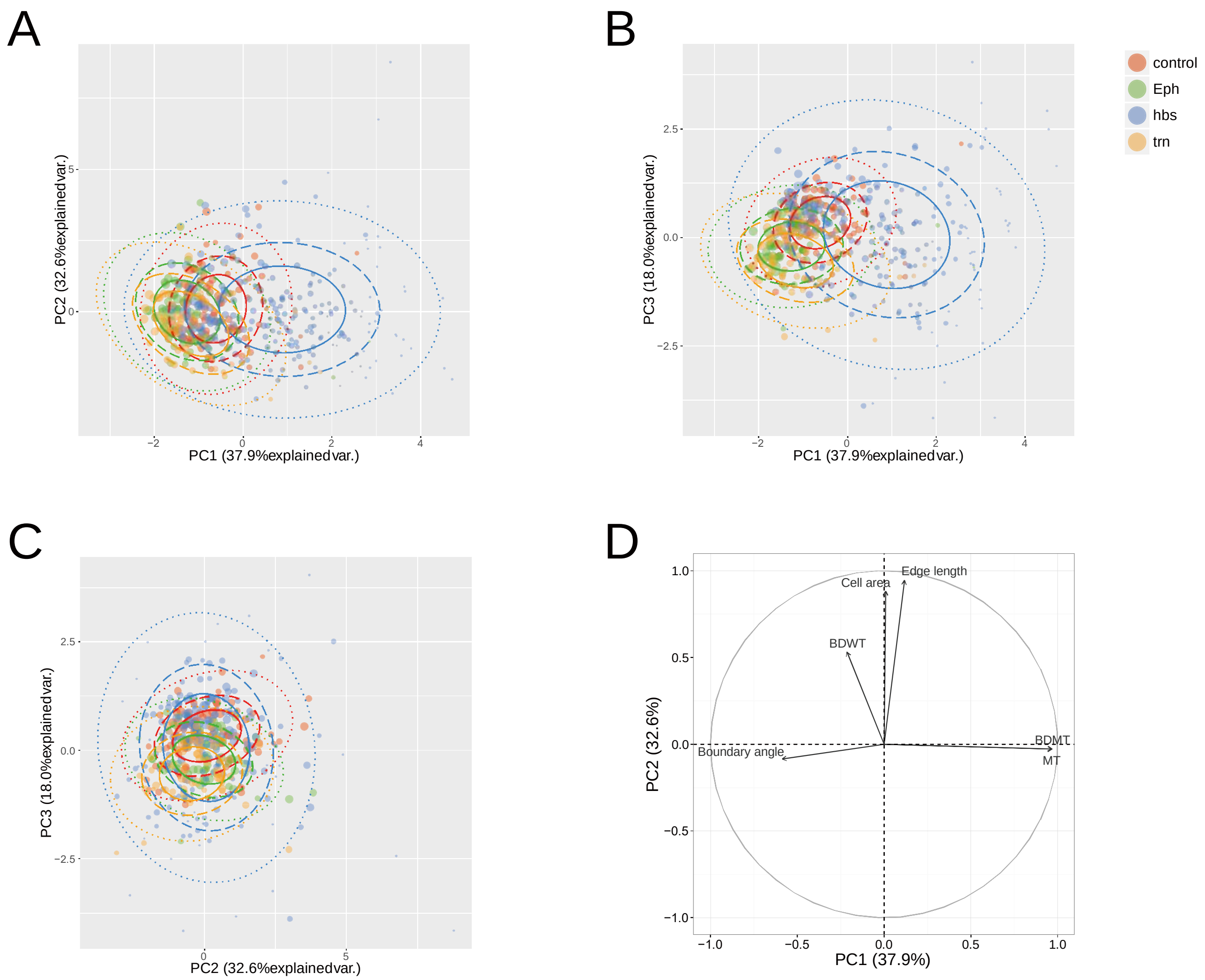

Supplementary Figure 6.
(A–C) PCA using the same six criteria shown in Figure 3H–J without averaging of individual clones within each wing disc. **(D)** The quantified contribution (factor loading) of each criterion on PC1 and PC2 in **(A)** was similar to that in Figure 3K.

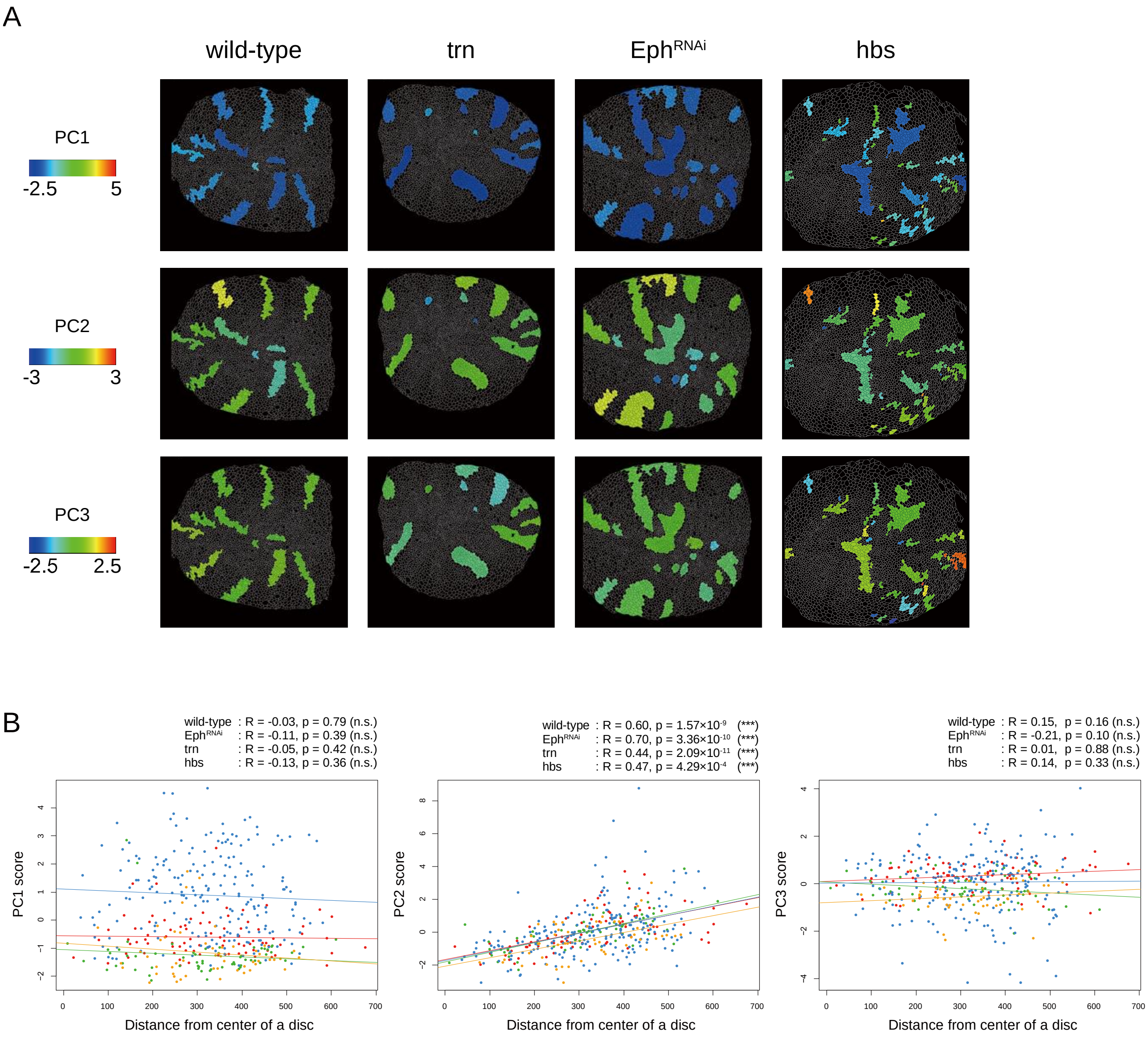

Supplementary Figure 7.

(A) Visualization of the scores of PC1, PC2, and PC3 for the examined clones of the four genotypes shown in Figure S6A–C. **(B)** The PC2 score has a positive correlation with the distance from the center of the disc (middle panel), while the PC1 and PC3 scores do not (left and right panels). The center position was determined manually by referring to the position of the highest E-cad intensity. Pearson correlation coefficient *R* and test for the association between paired samples were calculated using the R environment with the "cor" and "cor.test" functions.

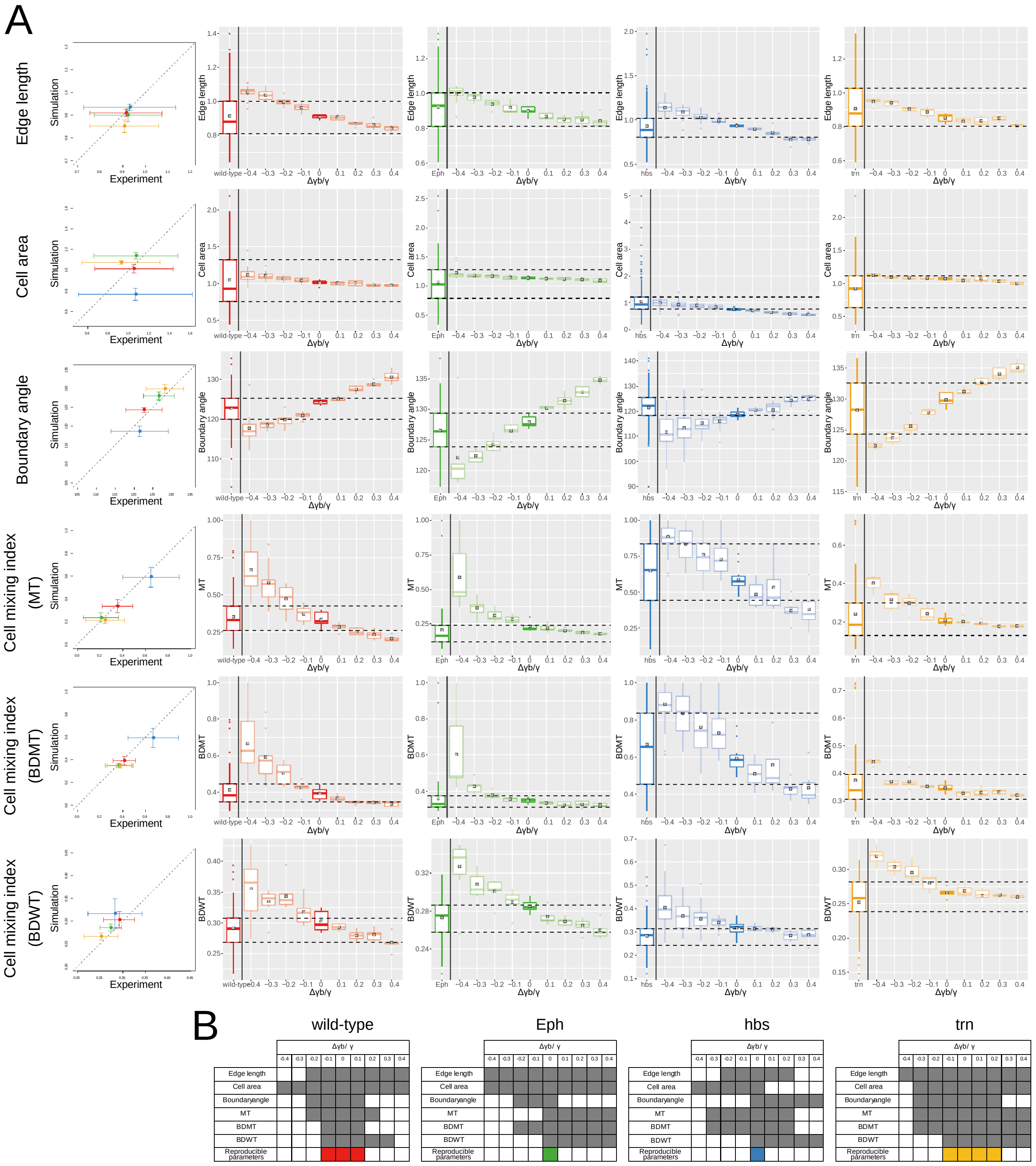

Supplementary Figure 8.

Evaluating precision of estimated line tension parameter γ_b. **(A)** Left most panels: Validation of the best representative parameters (γ_b and γ_c indicated by pentagon asterisks in Figure 5B) in simulation by comparing clone shape criteria with those in experiment. Wild-type (red), *Eph*-RNAi (green), *hbs*-overexpression (blue), *trn*-overexpression (yellow). Error bars of experiment were SD (standard deviations) of all clones which were identical datasets plotted in Figure S2, whereas that of simulation were SD of all clones at the estimated parameters (asterisks in Figure 5B). Distance

between the plots and y = x (dashed line) were less than SDs, indicating experiment was reproduced by the estimated parameters. Right panels (box plots) evaluated the parameter precision of γ_b for each genotype (horizontal direction) regarding six clone shape criteria (vertical direction). Box plot of each genotype was shown at the left most side for each panel indexed by "wild-type", "Eph", "hbs" and "trn", whose datasets were identical with left most panels. Black dashed lines denote 25th and 75th percentiles of the experimental plots. Box plots at the right side of black solid line show simulated clones as a function of parameter shift $\Delta \gamma_b/\gamma$ from the best representative parameter ($\Delta\gamma_b/\gamma = 0$). **(B)** Grey filled region denotes box plots in simulation overlapped with that of experiment bounded between black dashed lines in **A**. Colored region at the lowest row indexed by "reproducible parameters" summarizes the parameter shift which reproduced experiments in all criteria. The maxima of absolute value of the reproducible parameters (colored $\Delta\gamma_b/\gamma$) [±0.1 (wild-type), under ±0.1 (*Eph, hbs*), and ±0.2 (*trn*)] were defined as the estimation precision of γ_b/γ. The estimated precision of γ_b/γ are equal to that of clone tension regarding γ_b, based on Equation 2 in Main text.

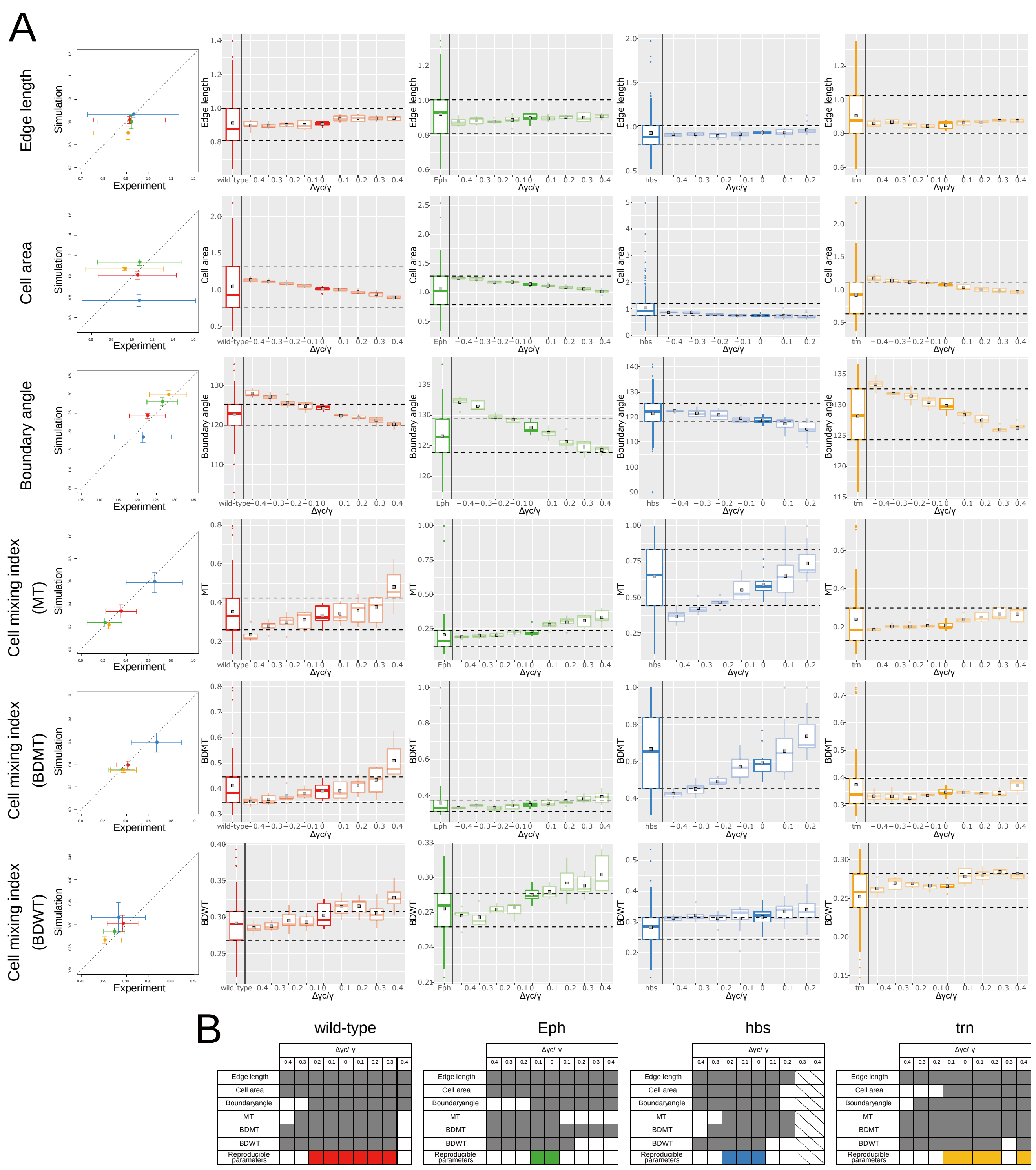

Supplementary Figure 9.

Evaluating precision of estimated line tension parameter γ_c. **(A-B)** The definition and layout of plots were the same with Supplementary Figure 8, except for evaluating the parameter precision of γ_c in right panels. We note that simulation parameter shifting +0.3 and +0.4 ($\Delta\gamma_c/\gamma$ = 0.3, 0.4) for *hbs* was absent, because the parameters were out of range. Following the evaluation of estimation precision of γ_b in Figure S8, precision estimation of γ_c was evaluated as ±0.1 (*Eph*), ±0.2 (*hbs*, *trn*), and ±0.3 (wild-type), while $\Delta\gamma_c/\gamma$ = 0.4 in *trn* was unlikely because simulation with $\Delta\gamma_c/\gamma$ = 0.3 already failed

to reproduce experiment. These values are twice of the estimation precision of clone tension regarding γ_c, calculated from Equation 2 in Main text. The estimation precision of clone tension was totally given by larger value between that regarding γ_b (Figure S8) and γ_c; ±0.15 (wild-type), under ±0.1 (*Eph*), ±0.1 (*hbs*), ±0.2 (*trn*).

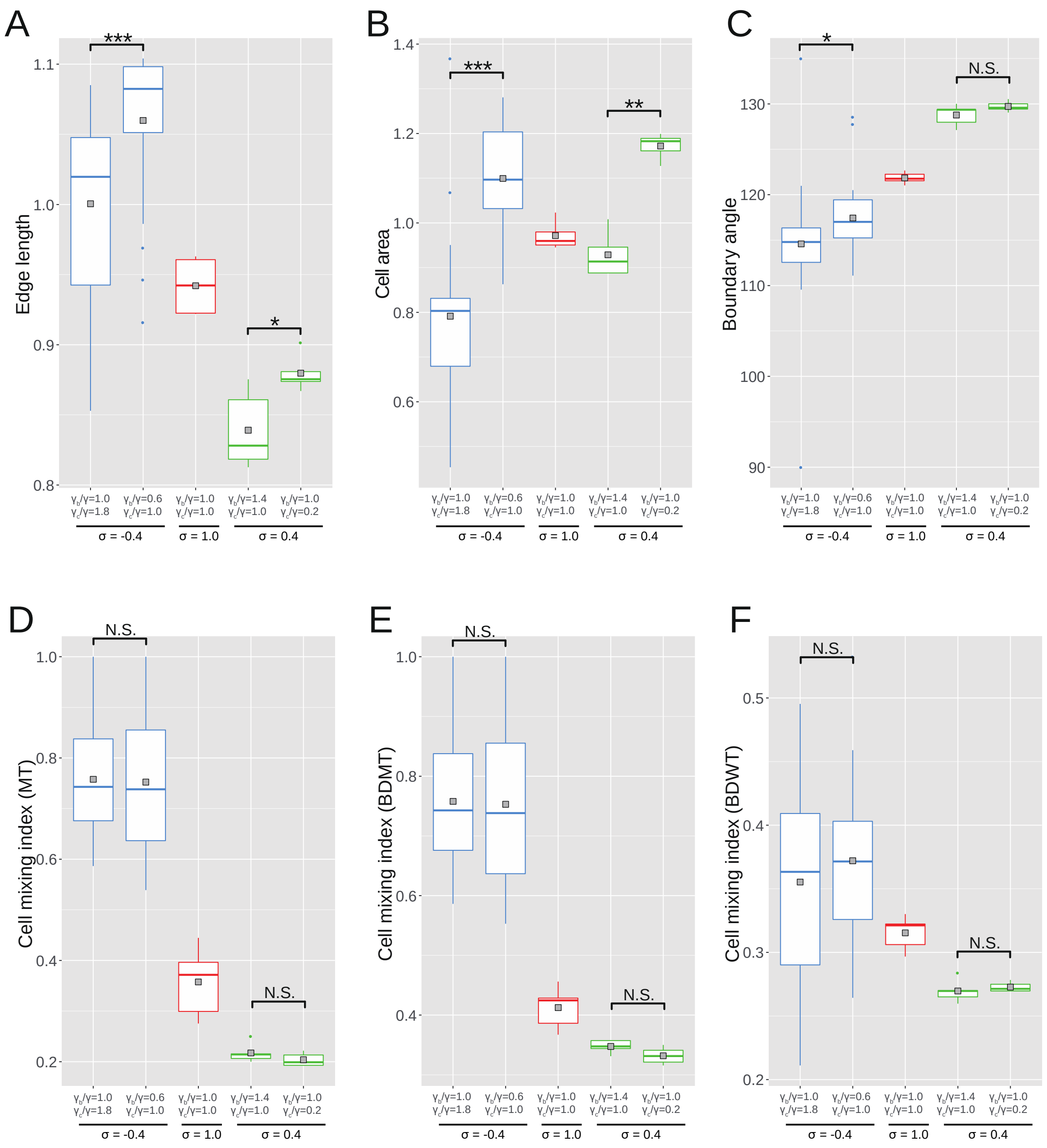

Supplementary Figure 10.

Role of mechanical parameters γ_b and γ_c on clone shape under constant clone tension σ in simulations (σ = -0.4 and 0.4; see Figure 4C for corresponding clone shape). Box plot of **(A)** Edge length, **(B)** cell area, **(C)** boundary angle, **(D)** MT, **(E)** BDMT and **(F)** BDWT (see Figure 1D-J for definition) for all clones at 40 cells in clones in total. Edge length and cell area were significantly smaller as γ_b and γ_c got larger at constant σ (σ = -0.4 and 0.4; A-B) while the other indicators showed mostly not significant difference (C-F). The physical reason of this difference is that larger line tension parameter γ_c contributes to shorten edge length so as to decrease cell area inside of the clone bulk. In addition, larger line tension parameter γ_b contributes to shorten edge length at the clone boundary. Wilcoxon rank sum test. n = 5 independent simulations for each parameter. $P < 0.001$ (***), $P < 0.01$ (**), $P < 0.05$ (*) and $P > 0.05$ (N.S., Not Significant).

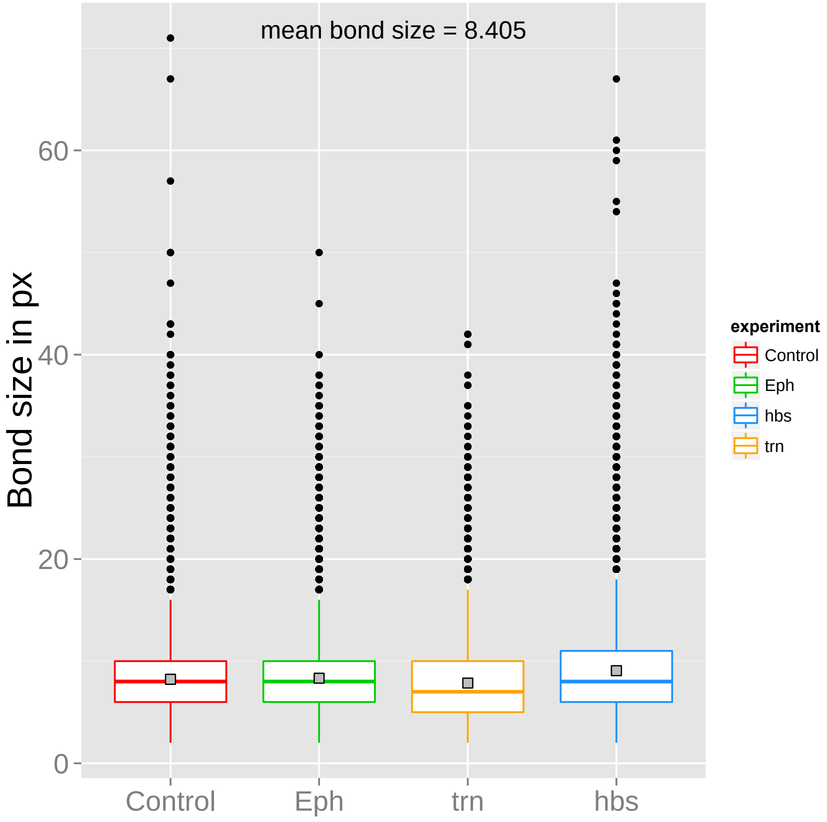

Supplementary Figure 11.

The mean bond size in pixel for each experiment. The mean bond size was calculated for all bonds including inside and outside of clones in all experiments. The data was plotted for each genotype separately. The mean bond size for all cell bonds of all experiments was 8.4 pixels/bond.

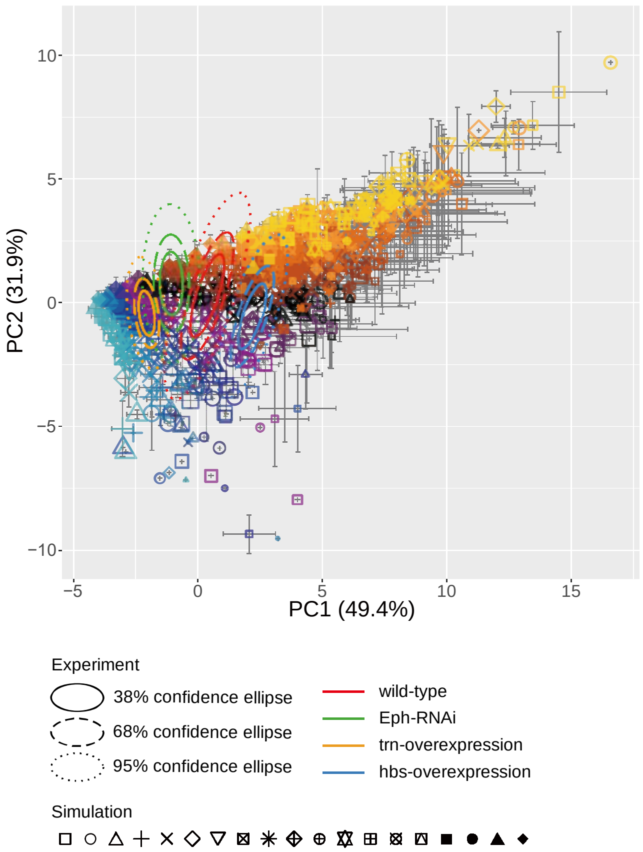

Supplementary Figure 12.
Projection of all simulated clones onto the PC space of *Drosophila* experiments, which is a complete version of Figure 5A.

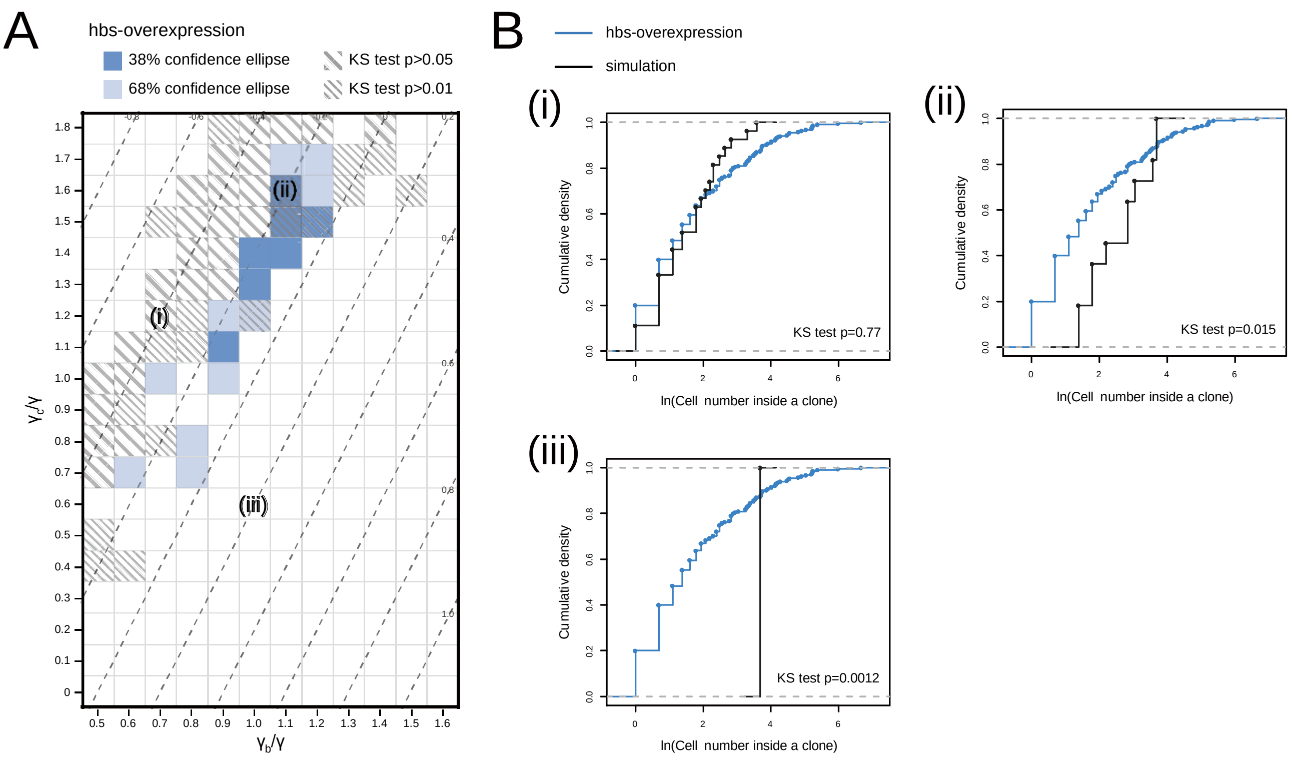

Supplementary Figure 13.

(A) The parameter region of *hbs* was limited to a more narrow range by combining it with another observation criterion, the distribution of cell number within a clone **(B)**. In the regions containing grey oblique lines, the empirical cumulative distribution of the cell number within clones was similar to that of *hbs*-overexpression clones (KS test, $P > 0.05$ or $P > 0.01$). The blue-colored regions showing the estimated mechanical parameters of *hbs*-overexpression clones generated by projection onto the PC space were the same as those in Figure 5B. The ranges of the grey-oblique and both blue-colored regions were partially overlapped each other. **(B)** The empirical cumulative distribution of cell number within clones was compared between *hbs*-overexpression clones and simulated clones [(i) $\gamma_b/\gamma = 0.7$, $\gamma_c/\gamma = 1.2$, (ii) $\gamma_b/\gamma = 1.1$, $\gamma_c/\gamma = 1.6$, (iii) $\gamma_b/\gamma = 1.0$, $\gamma_c/\gamma = 0.6$].

	Edge length	Cell area	Boundary angle	MT	BDMT	BDWT
Weight (PC1)	0.153	0.205	-0.519	0.525	0.505	0.366
Weight (PC2)	0.630	0.635	0.122	-0.248	-0.247	0.251
Weight (PC3)	0.367	0.134	0.259	0.258	0.380	-0.754

Supplementary Table 1.
Weights for each criteria to calculate PC scores in Figure 3H-K, which correspond to a_{11}, a_{12}, …, a_{16} in Section Projection of the Simulation Data onto the PCA Space of the Experimental Data in Main Text.
